# Crystal structures of [*Ln*(NO_3_)_3_(μ_2_-bpydo)_2_], where *Ln* = Ce, Pr or Nd, and bpydo = 4,4′-bi­pyridine *N*,*N*′-dioxide: layered coordination networks containing 4^4^ grids

**DOI:** 10.1107/S205698901502318X

**Published:** 2016-01-01

**Authors:** Michael L. Stromyer, Cassandra P. Lilly, Adam J. Dillner, Jacqueline M. Knaust

**Affiliations:** a Department of Chemistry Mathematics and Physics, Clarion University, 840 Wood Street, Clarion, PA 16214, USA; b Chemistry Department, 520 North Main St, Meadville, PA 16335, USA

**Keywords:** crystal structure, cerium coordination network, praseodymium coordination network, neodymium coordination network, 4,4′-bi­pyridine *N*,*N′*-dioxide (bpydo), C—H⋯O inter­actions, π–π inter­actions, 4^4^ grid

## Abstract

Three isostructural coordination networks of Ce, Pr, and Nd nitrate with 4,4′-bi­pyridine *N*,*N*′-dioxide (bpydo) are reported, namely poly[[tris­(nitrato-κ^2^ *O*,*O*′)cerium(III)]-bis­(μ_2_-4,4′-bi­pyridine *N*,*N*′-dioxide-κ^2^ *N*:*N*′)], [Ce(NO_3_)_3_(C_10_H_8_N_2_O_2_)_2_], poly[[tris­(nitrato-κ^2^ *O*,*O*′)praeseodymium(III)]-bis­(μ_2_-4,4′-bi­pyridine *N*,*N*′-dioxide-κ^2^ *N*:*N*′)], [Pr(NO_3_)_3_(C_10_H_8_N_2_O_2_)_2_], and poly[[tris(nitrato-κ^2^ *O*,*O*′)neodymium(III)]-bis­(μ_2_-4,4′-bi­pyridine *N*,*N*′-dioxide-κ^2^ *N*:*N*′], [Nd(NO_3_)_3_(C_10_H_8_N_2_O_2_)_2_]. All three compounds are isostructural to the previously reported La analogue. The asymmetric unit of [*Ln*(NO_3_)_3_(μ_2_-bpydo)_2_] contains one lanthanide cation, two bpydo ligands, and three nitrate anions. Both bpydo ligands act as end-to-end μ_2_-bridges and display nearly ideal *cis* and *gauche* conformations, respectively. The bpydo ligands link the ten-coordinate *Ln* ^III^ cations, forming inter­digitating 4^4^ grid-like layers extending parallel to (-101), where inter­digitation of layers is promoted by C—H⋯O inter­actions between nitrate anions and bpydo ligands. The inter­digitated layers are linked to sets of neighboring layers *via* further C—H⋯O and π–π inter­actions.

## Abstract 2

The crystal structures of poly[bis­(μ_2_-4,4′-bi­pyridine *N*,*N*′-dioxide-κ^2^ *O*:*O′*)trinitratocerium(III)] and its isostructural praseodymium and neodymium analogues feature a 4^4^ grid-like layered structure with inter­digitation of layers promoted by C—H⋯O inter­actions between nitrate anions and 4,4′-bi­pyridine *N*,*N*′-dioxide ligands.

## Chemical context   

The use of aromatic *N,N′*-dioxide ligands such as 4,4′-bi­pyridine *N*,*N*′-dioxide (bpydo) in the synthesis of lanthanide compounds comprising coordination networks has been of recent inter­est (Dillner *et al.*, 2010*a* ▸,*b* ▸; Hill *et al.*, 2004 ▸, 2005*a* ▸,*b* ▸; Long *et al.*, 2000 ▸, 2002 ▸). The coordination modes of aromatic *N,N′*-dioxide ligands are flexible; they may act as terminal ligands, end-on or end-to-end μ_2_-bridges, μ_3_-bridges, or μ_4_-bridges (Lu *et al.*, 2002 ▸; Ma *et al.*, 2001 ▸, 2003 ▸; Zhang *et al.*, 2004*a* ▸,*b* ▸). When acting as end-to-end μ_2_-bridges, these ligands can display *cis*, *gauche*, or *trans* conformations where the ideal conformations have *M*—O⋯O—*M* torsion angles of 0, 90 and 180°, respectively (Sun *et al.*, 2004 ▸). Furthermore, aromatic *N,N′*-dioxide ligands are able to participate in a variety of hydrogen-bonding inter­actions (González Mantero *et al.*, 2006 ▸). Structure prediction with these ligands can be difficult, not only due to their flexible bonding modes and various hydrogen-bonding inter­actions, but also due to the influences of solvent and anion (Hill *et al.*, 2005*a* ▸).

## Structural commentary   

Three isostructural coordination networks of Ce, Pr, and Nd nitrate with 4,4′-bi­pyridine *N*,*N*′-dioxide (bpydo), [*Ln*(NO_3_)_3_(μ_2_-bpydo)_2_] [*Ln* = Ce (I), Pr (II), and Nd (III)] are reported. All three compounds are isostructural to the previously reported La analogue (Hill *et al.*, 2004 ▸). 
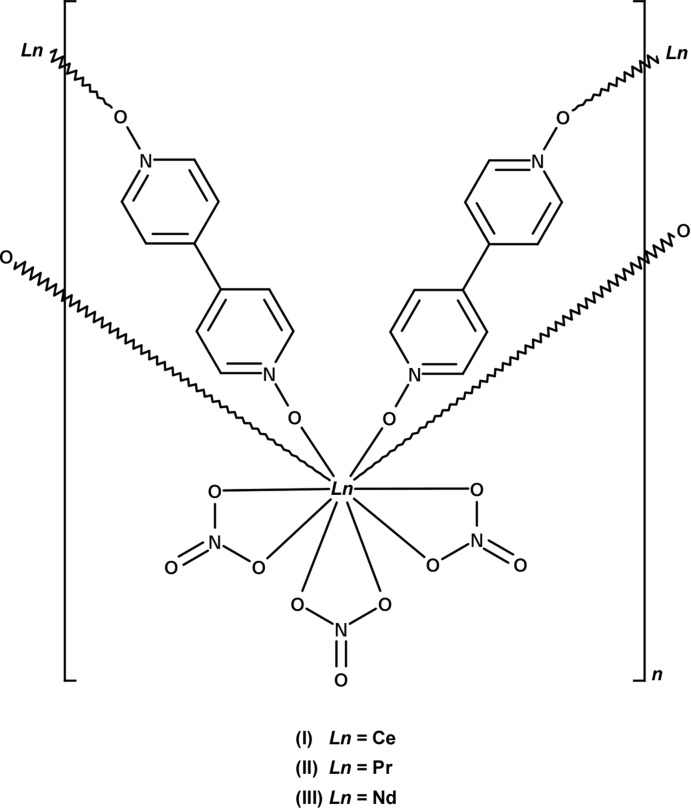


**Figure 1:**
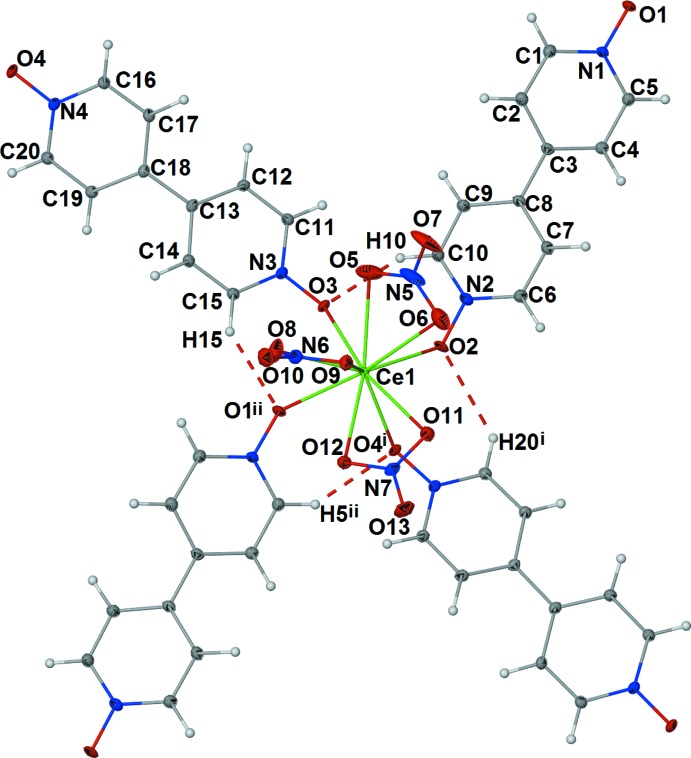
Coordination sphere around the Ce^III^ cation in the structure of (I), with displacement ellipsoids drawn at the 50% probability level. Dashed lines represent C—H⋯O inter­actions between neighboring bpydo ligands within the coordination sphere. [Symmetry codes: (i) *x* − 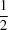, −*y* + 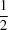, *z* − 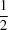; (ii) *x*, *y* − 1, *z*.]

**Figure 2:**
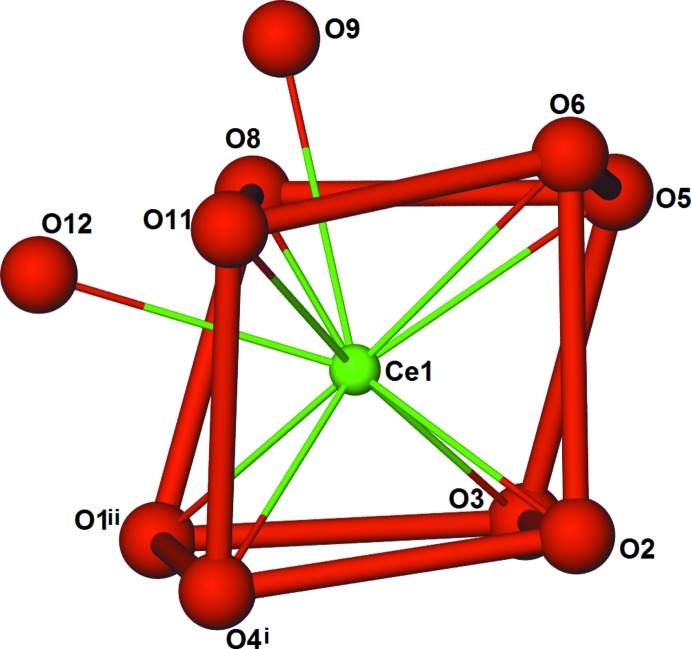
*Ln*O_10_ coordination environment forming a distorted bicapped square prism. [Symmetry codes: (i) *x* − 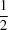, −*y* + 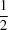, *z* − 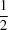; (ii) *x*, *y* − 1, *z*.]

**Figure 3:**
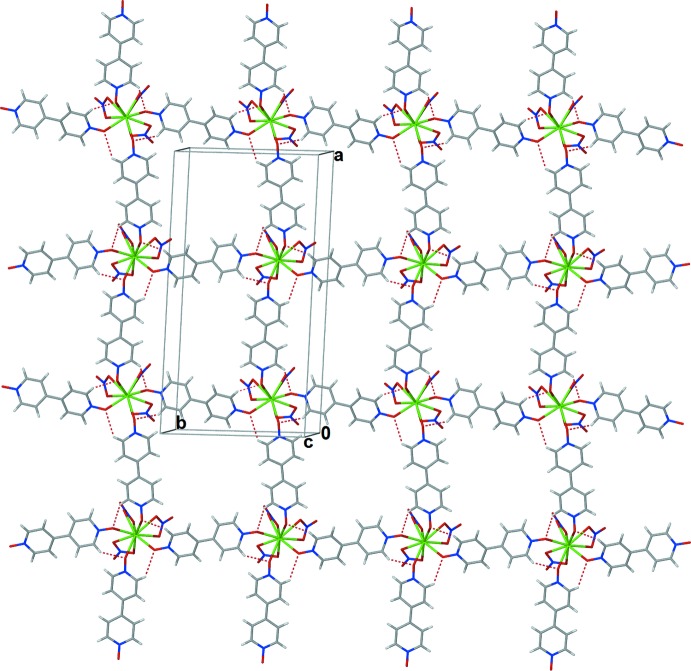
Diagram showing the 4^4^ grid-like layers that lie parallel to (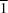
01) in (I). Dashed lines represent C—H⋯O inter­actions between neighboring bpydo ligands within the Ce^III^ coordination sphere.

**Figure 4:**
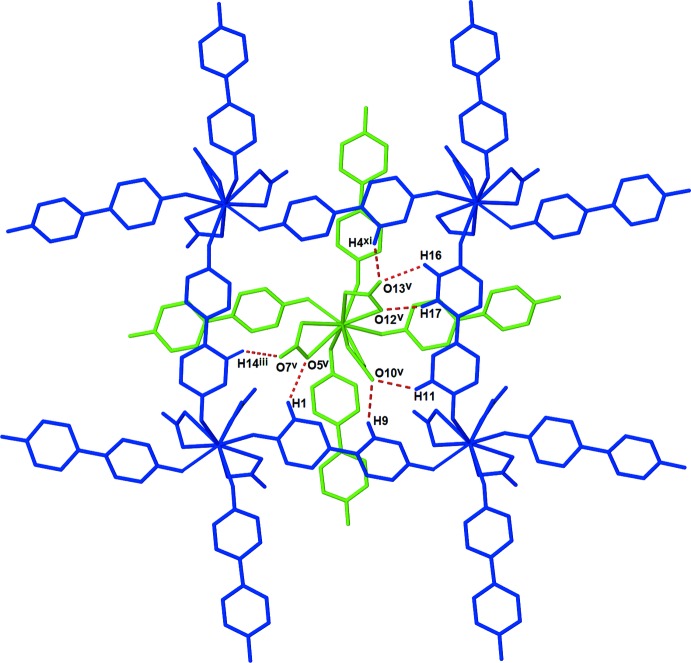
Diagram showing the C—H⋯O inter­actions between anions and ligands of inter­digitated layers in (I). Individual layers are represented in green and blue. Dashed red lines represent C—H⋯O inter­actions between the layers. [Symmetry codes: (iii) *x*, *y* + 1, *z*; (v) −*x* + 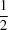, *y* + 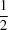, −*z* + 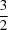; (xi) *x* + 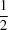, −*y* + 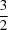, *z* + 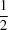.]

The asymmetric unit of [*Ln*(NO_3_)_3_(μ_2_-bpydo)_2_] contains one lanthanide cation, two end-to-end bridging μ_2_-bpydo ligands, and three chelating nitrate anions. All atoms in the asymmetric unit lie on general positions (Fig. 1 ▸). The *Ln* ^III^ atoms have a coordination sphere defined by six oxygen atoms from chelating nitrate anions and four oxygen atoms from bpydo ligands. The ten oxygen atoms in the *Ln*O_10_ coordination environment form a distorted bi-capped square prism (Fig. 2 ▸). One of the ligands bridges in a nearly perfect *cis* conformation with an *Ln*l—O3⋯O4—*Ln*1^iv^ torsion angle of approximately 5° and a dihedral angle between the rings of approximately 33°. The other ligand bridges in a nearly perfect *gauche* conformation with an *Ln*l—O2⋯O1—*Ln*1^iii^ torsion angle of approximately 92° and a dihedral angle between the rings of approximately 28° (see Table 1 ▸). The bpydo ligands link the *Ln* ^III^ atoms, forming 4^4^ grid-like layers that are parallel to (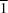
01) (Fig. 3 ▸). Each layer inter­digitates with a symmetry-equivalent second layer related by a twofold screw axis. The nitrate anions chelate to the metal cations on one side of the 4^4^ grid and are directed towards the square void of the symmetry-related inter­digitated 4^4^ grid (Fig. 4 ▸).

While a roughly linear decrease in cell volume for a series of isostructural lanthanide compounds due to the lanthanide contraction may be expected (see, for example, He *et al.*, 2005 ▸; Ji *et al.*, 2012 ▸), deviations from a linear trend as observed for compounds (I)–(III) are not unprecedented, and the gradual decrease in *Ln*—*X* bond lengths and bridged *Ln*⋯*Ln* distances provides evidence of the lanthanide contraction (see, for example, Jia *et al.*, 2013 ▸; Li *et al.*, 2004 ▸, 2015 ▸). Recent studies on several series of isostructural lanthanide compounds have shown that the lanthanide contraction can be observed by the quadratic decay of the *Ln*—O bond lengths with increasing atomic number (Quadrelli, 2002 ▸; Seitz *et al.*, 2007 ▸; Xu *et al.*, 2013 ▸). An examination of both the *Ln*—O_bpydo_ and *Ln*—O_nitrate_ distances for compounds (I)–(III) shows the expected gradual decrease in the *Ln*—O bond lengths from Ce (I) to Nd (III) due to the lanthanide contraction (Table 2 ▸). The gradual decrease in bpydo-bridged *Ln*⋯*Ln* distances within the layers is also consistent with the radius contraction from Ce to Nd (Table 1 ▸).

## Supra­molecular features   

**Figure 5:**
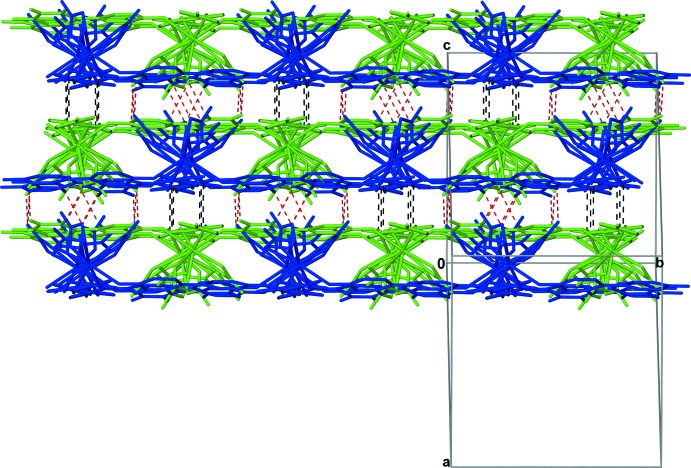
Diagram showing C—H⋯O inter­actions and π–π inter­actions that link each set of inter­digitated layers to similar sets of layers above and below it in (I). Individual layers are represented in green and blue. Dashed red lines represent C—H⋯O inter­actions, and dashed black lines represent π–π inter­actions.

Stabilizing C—H⋯O inter­actions (C5—H5⋯O4^vii^, C10—H10⋯O3, C15—H15⋯O1^ii^, and C20—H20⋯O2^iv^) are observed between neighboring bpydo ligands within the coordination sphere of the *Ln* ^III^ cation (see Tables 3 ▸–5 ▸ ▸ for symmetry codes; Fig. 1 ▸). The inter­digitation of layers is promoted by C—H⋯O inter­actions (C1—H1⋯O5^v^, C4—H4⋯O13^vi^, C9—H9⋯O10^v^, C11—H11⋯O10^v^, C14—H14⋯O7^ix^, C16—H16⋯O13^v^, and C17—H17⋯O12^v^) between the ligands of one layer and nitrate anions of the other layer (Fig. 4 ▸). Further C—H⋯O inter­actions (C9—H9⋯O9^viii^ and C10—H10⋯O7^viii^) and π–π inter­actions between *Cg*1 and the inversion-related *Cg*1^x^ link each set of inter­digitated layers to symmetry-equivalent sets of layers above and below it [symmetry code: (x) −*x* + 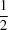, −*y* + 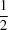, −*z* + 2; Fig. 5 ▸). π–π inter­actions between the neighboring rings are observed with a centroid-to-centroid distance of 3.7535 (10) Å and an inter­planar distance of 3.2830 (6) Å for (I); there is a slippage of 1.820 Å such that H15^x^ of the neighboring *N*-oxide ring lies nearly centered over the centroid of *Cg*1 at a distance of 3.305 Å [see Table 1 ▸ for distances in compounds (II) and (III)].

## Database survey   

A survey of the Cambridge Structural Database (CSD, November 2014; Groom & Allen, 2014 ▸) returned hits for 333 structures with 4,4′-bi­pyridine *N*,*N′*-dioxide. Sixty three structures are reported where bpydo coordinates to a lanthanide metal and acts a as bridging ligand in a coordination network. Of these structures, ten are reported with nitrate as the counter-ion. In [Tb(bpydo)_2_(NO_3_)_3_], linear chains are observed (Long *et al.*, 2002 ▸). A one-dimensional network composed of zigzag chains is observed for [Tb(bpydo)(CH_3_OH)(NO_3_)_3_] (Long *et al.*, 2002 ▸). In {[Ln(bpydo)_1.5_(NO_3_)_3_]·CH_2_Cl_2_} with *Ln* = Eu (Dillner *et al.*, 2010*a* ▸), Gd (Dillner *et al.*, 2010*b* ▸), and Tb (Long *et al.*, 2002 ▸), a one-dimensional network composed of ladder-like chains is observed. [La(bpydo)_2_(NO_3_)_3_] is a two-dimensional network composed of sheets with 4^4^ topology and is isostructural to the Ce, Pr, and Nd structures reported herein (Hill *et al.*, 2004 ▸). In {[Er_2_(bpydo)_3_(NO_3_)_6_]·2CH_3_OH}, {[Tb(bpydo)_1.5_(NO_3_)_3_]·CH_3_OH·0.8H_2_O}, and {[Tb(bpydo)_1.5_(NO_3_)_3_]·0.4CCl_4_·0.8CH_3_OH}, two-dimensional networks composed of sheets with 4.8^2^ topology are formed (Long *et al.*, 2000 ▸, 2002 ▸). In {[Sm(bpydo)_2_(NO_3_)_3_]·0.5H_2_O}, a twofold inter­penetrating three-dimensional network is formed (Long *et al.*, 2000 ▸).

## Synthesis and crystallization   

4,4′-bi­pyridine *N*,*N′*-dioxide·H_2_O was synthesized from 4,4′-bi­pyridine according to the method of Simpson *et al.* (1963 ▸). All other chemicals were obtained from commercial sources and used without further purification. For the Ce, Pr and Nd compounds, respectively, the appropriate *Ln*(NO_3_)_3_·6H_2_O (0.113 mmol) was placed in the bottom of a test tube and covered with CH_2_Cl_2_ (5 ml). 4,4′-Bi­pyridine-*N*,*N*′-dioxide·H_2_O (0.0376 g, 0.182 mmol) was dissolved in methanol (8 ml), and this solution was layered over the CH_2_Cl_2_ solution. The two solutions were allowed to slowly mix. Over a period of several weeks the *Ln*(NO_3_)_3_·6H_2_O dissolved, and red block-like crystals of [Ce(μ_2_-bpydo)_2_(NO_3_)_3_], yellow block-like crystals of [Pr(μ_2_-bpydo)_2_(NO_3_)_3_], and yellow block-like crystals of [Nd(μ_2_-bpydo)_2_(NO_3_)_3_] were formed.

## Refinement   

All aromatic H atoms were positioned geometrically and refined using a riding model with C—H = 0.95 Å and with *U*_iso_(H) = 1.2*U*_eq_(C). Crystal data, data collection and structure refinement details are summarized in Table 6 ▸.

## Supplementary Material

Crystal structure: contains datablock(s) global, I, II, III. DOI: 10.1107/S205698901502318X/wm5242sup1.cif

Structure factors: contains datablock(s) I. DOI: 10.1107/S205698901502318X/wm5242Isup2.hkl

Structure factors: contains datablock(s) II. DOI: 10.1107/S205698901502318X/wm5242IIsup3.hkl

Structure factors: contains datablock(s) III. DOI: 10.1107/S205698901502318X/wm5242IIIsup4.hkl

CCDC references: 1440109, 1440108, 1440107

Additional supporting information:  crystallographic information; 3D view; checkCIF report

## Figures and Tables

**Table 1:** Selected geometric parameters (Å, °) for (I)–(III) Dihedral angles are reported between the mean planes defined by the indicated aromatic rings. *Cg*1 is the centroid of the N3/C11–C15 ring.

		(I)	(II)	(III)
*Ln*⋯*Ln* distances				
	*Ln*1⋯*Ln*1^iii^	13.3398 (13)	13.3127 (9)	13.3035 (5)
	*Ln*1⋯*Ln*1^iv^	13.2996 (11)	13.2634 (8)	13.2558 (4)
Dihedral angles				
	N1/C1–C5⋯N2/C6–C10	27.387 (58)	28.041 (62)	28.471 (109)
	N3/C11–C15⋯N4/C16–C20	22.560 (50)	22.552 (55)	22.677 (93)
Torsion angles				
	*Ln*1—O2⋯O1—*Ln*1^iii^	92.53 (6)	91.80 (6)	91.75 (11)
	*Ln*1—O3⋯O4—*Ln*1^iv^	5.38 (7)	4.86 (8)	4.87 (14)
π–π inter­actions for *Cg*1⋯*Cg*1^*x*^				
	Centroid–centroid distance	3.7535 (10)	3.7465 (10)	3.7344 (17)
	Inter­planar distance	3.2830 (6)	3.2790 (7)	3.2815 (11)
	Slippage	1.820	1.810	1.783
	*Cg*1—H15^*x*^ distance	3.305	3.312	3.311

Symmetry codes: (iii) *x*, *y* + 1, *z*; (iv) *x* + 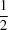, −*y* + 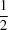, *z* + 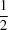; (*x*) −*x* + 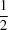, −*y* + 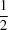, −*z* + 2.

**Table 2:** Selected bond lengths (Å) in compounds (I)–(III)

	Compound	(I)	(II)	(III)
*Ln*—O bond lengths involving bpydo ligands				
	*Ln*1—O1^ii^	2.5464 (11)	2.5360 (12)	2.526 (2)
	*Ln*1—O2	2.5192 (11)	2.5009 (12)	2.488 (2)
	*Ln*1—O3	2.4685 (11)	2.4558 (11)	2.451 (2)
	*Ln*1—O4^i^	2.4692 (11)	2.4554 (12)	2.448 (2)
	Average *Ln*—O distances	2.501	2.487	2.478
*Ln*—O bond lengths involving chelating nitrate anions				
	*Ln*1—O5	2.5929 (13)	2.5750 (13)	2.555 (2)
	*Ln*1—O6	2.6573 (13)	2.6443 (14)	2.640 (2)
	*Ln*1—O8	2.6004 (12)	2.5832 (13)	2.573 (2)
	*Ln*1—O9	2.6428 (12)	2.6242 (13)	2.615 (2)
	*Ln*1—O11	2.6231 (12)	2.6036 (12)	2.585 (2)
	*Ln*1—O12	2.6333 (11)	2.6147 (12)	2.597 (2)
	Average *Ln*—O distances	2.625	2.608	2.594

Symmetry codes: (i) *x* − 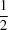, −*y* + 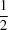, *z* − 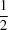; (ii) *x*, *y* − 1, *z*.

**Table 3:** Hydrogen-bond geometry (Å, °) for (I)

*D*—H⋯*A*	*D*—H	H⋯*A*	*D*⋯*A*	*D*—H⋯*A*
C1—H1⋯O5^v^	0.95	2.59	3.342 (2)	136
C4—H4⋯O13^vi^	0.95	2.37	3.208 (2)	148
C5—H5⋯O4^vii^	0.95	2.38	3.1868 (19)	142
C9—H9⋯O9^viii^	0.95	2.62	3.206 (2)	121
C9—H9⋯O10^v^	0.95	2.59	3.475 (2)	156
C10—H10⋯O3	0.95	2.32	3.128 (2)	143
C10—H10⋯O7^viii^	0.95	2.58	3.264 (2)	129
C11—H11⋯O10^v^	0.95	2.49	3.237 (2)	135
C14—H14⋯O7^ix^	0.95	2.22	3.004 (2)	139
C15—H15⋯O1^ii^	0.95	2.32	3.1069 (19)	140
C16—H16⋯O13^v^	0.95	2.55	3.154 (2)	122
C17—H17⋯O12^v^	0.95	2.36	3.2837 (19)	164
C20—H20⋯O2^iv^	0.95	2.63	3.3265 (19)	130

Symmetry codes: (ii) 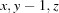; (iv) 
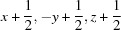
; (v) 
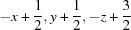
; (vi) 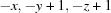; (vii) 
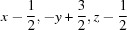
; (viii) 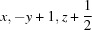; (ix) 
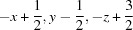
.

**Table 4:** Hydrogen-bond geometry (Å, °) for (II)

*D*—H⋯*A*	*D*—H	H⋯*A*	*D*⋯*A*	*D*—H⋯*A*
C1—H1⋯O5^v^	0.95	2.59	3.331 (2)	135
C4—H4⋯O13^vi^	0.95	2.36	3.200 (2)	147
C5—H5⋯O4^vii^	0.95	2.37	3.168 (2)	141
C9—H9⋯O9^viii^	0.95	2.61	3.204 (2)	121
C9—H9⋯O10^v^	0.95	2.58	3.468 (2)	156
C10—H10⋯O3	0.95	2.31	3.115 (2)	143
C10—H10⋯O7^viii^	0.95	2.60	3.277 (3)	129
C11—H11⋯O10^v^	0.95	2.50	3.239 (2)	135
C14—H14⋯O7^ix^	0.95	2.22	3.002 (2)	139
C15—H15⋯O1^ii^	0.95	2.31	3.0924 (19)	140
C16—H16⋯O13^v^	0.95	2.56	3.154 (2)	121
C17—H17⋯O12^v^	0.95	2.36	3.288 (2)	164
C20—H20⋯O2^iv^	0.95	2.62	3.307 (2)	130

Symmetry codes: (ii) 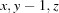; (iv) 
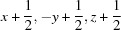
; (v) 
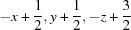
; (vi) 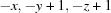; (vii) 
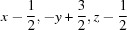
; (viii) 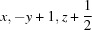; (ix) 
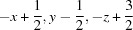
.

**Table 5:** Hydrogen-bond geometry (Å, °) for (III)

*D*—H⋯*A*	*D*—H	H⋯*A*	*D*⋯*A*	*D*—H⋯*A*
C1—H1⋯O5^v^	0.95	2.61	3.353 (4)	135
C4—H4⋯O13^vi^	0.95	2.37	3.206 (4)	147
C5—H5⋯O4^vii^	0.95	2.37	3.163 (4)	141
C9—H9⋯O9^viii^	0.95	2.63	3.216 (4)	121
C9—H9⋯O10^v^	0.95	2.58	3.464 (4)	156
C10—H10⋯O3	0.95	2.30	3.110 (4)	142
C10—H10⋯O7^viii^	0.95	2.61	3.289 (4)	129
C11—H11⋯O10^v^	0.95	2.50	3.243 (4)	135
C14—H14⋯O7^ix^	0.95	2.21	2.998 (4)	139
C15—H15⋯O1^ii^	0.95	2.31	3.091 (4)	139
C16—H16⋯O13^v^	0.95	2.56	3.159 (4)	121
C17—H17⋯O12^v^	0.95	2.37	3.295 (4)	165
C20—H20⋯O2^iv^	0.95	2.61	3.294 (4)	130

Symmetry codes: (ii) 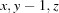; (iv) 
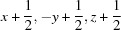
; (v) 
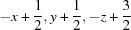
; (vi) 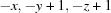; (vii) 
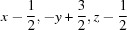
; (viii) 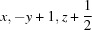; (ix) 
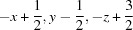
.

**Table 6:** Experimental details

	(I)	(II)	(III)
Crystal data			
Chemical formula	[Ce(NO_3_)_3_(C_10_H_8_N_2_O_2_)_2_]	[Pr(NO_3_)_3_(C_10_H_8_N_2_O_2_)_2_]	[Nd(NO_3_)_3_(C_10_H_8_N_2_O_2_)_2_]
*M*_r_	702.52	703.31	706.64
Crystal system, space group	Monoclinic, *C*2/*c*	Monoclinic, *C*2/*c*	Monoclinic, *C*2/*c*
Temperature (K)	173	173	173
*a*, *b*, *c* (Å)	26.786 (3), 13.3398 (13), 13.7571 (13)	26.7416 (18), 13.3127 (9), 13.7586 (9)	26.7422 (10), 13.3035 (5), 13.7804 (5)
β (°)	105.837 (1)	105.981 (1)	106.065 (1)
*V* (Å^3^)	4729.1 (8)	4708.8 (5)	4711.1 (3)
*Z*	8	8	8
Radiation type	Mo *K*α	Mo *K*α	Mo *K*α
μ (mm^−1^)	2.01	2.16	2.29
Crystal size (mm)	0.55 × 0.45 × 0.38	0.55 × 0.37 × 0.26	0.14 × 0.12 × 0.08

Data collection			
Diffractometer	Bruker APEXII CCD	Bruker APEXII CCD	Bruker D8 Quest CMOS
Absorption correction	Multi-scan (*SADABS*; Bruker, 2009 ▸)	Multi-scan (*SADABS*; Bruker, 2009 ▸ ▸)	Multi-scan (*SADABS*; Bruker, 2009 ▸ ▸)
*T*_min_, *T*_max_	0.536, 0.746	0.579, 0.746	0.682, 0.747
No. of measured, independent and observed [*I* > 2σ(*I*)] reflections	15990, 7152, 6686	18363, 7241, 6782	47148, 8277, 5419
*R*_int_	0.018	0.020	0.115
(sin θ/λ)_max_ (Å^−1^)	0.735	0.737	0.777

Refinement			
*R*[*F* ^2^ > 2σ(*F* ^2^)], *wR*(*F* ^2^), *S*	0.020, 0.050, 1.05	0.021, 0.052, 1.05	0.051, 0.067, 1.01
No. of reflections	7152	7241	8277
No. of parameters	370	370	370
H-atom treatment	H-atom parameters constrained	H-atom parameters constrained	H-atom parameters constrained
Δρ_max_, Δρ_min_ (e Å^−3^)	1.10, −0.65	0.89, −1.06	1.49, −1.29

Computer programs: *APEX2* and *SAINT* (Bruker, 2009 ▸, 2014 ▸), *SHELXS97* (Sheldrick, 2008 ▸), *SHELXL2014* (Sheldrick, 2015 ▸) and *X-SEED* (Barbour, 2001 ▸).

## supplementary crystallographic information

### (I) Poly[[tris(nitrato-κ2O,O')cerium(III)]-bis(µ-4,4'-bipyridine N,N'-dioxide-κ2N:N')] Crystal data

**Table:**

[Ce(NO_3_)_3_(C_10_H_8_N_2_O_2_)_2_]	*F*(000) = 2776
*M**_r_* = 702.52	*D*_x_ = 1.973 Mg m^−^^3^
Monoclinic, *C*2/*c*	Mo *K*α radiation, λ = 0.71073 Å
*a* = 26.786 (3) Å	Cell parameters from 11055 reflections
*b* = 13.3398 (13) Å	θ = 2.5–31.5°
*c* = 13.7571 (13) Å	µ = 2.01 mm^−^^1^
β = 105.837 (1)°	*T* = 173 K
*V* = 4729.1 (8) Å^3^	Block, red
*Z* = 8	0.55 × 0.45 × 0.38 mm

### (I) Poly[[tris(nitrato-κ2O,O')cerium(III)]-bis(µ-4,4'-bipyridine N,N'-dioxide-κ2N:N')] Data collection

**Table:**

Bruker APEXII CCD diffractometer	6686 reflections with *I* > 2σ(*I*)
phi and ω scans	*R*_int_ = 0.018
Absorption correction: multi-scan (SADABS; Bruker, 2009)	θ_max_ = 31.5°, θ_min_ = 1.6°
*T*_min_ = 0.536, *T*_max_ = 0.746	*h* = −37→37
15990 measured reflections	*k* = −11→19
7152 independent reflections	*l* = −20→19

### (I) Poly[[tris(nitrato-κ2O,O')cerium(III)]-bis(µ-4,4'-bipyridine N,N'-dioxide-κ2N:N')] Refinement

**Table:**

Refinement on *F*^2^	0 restraints
Least-squares matrix: full	Hydrogen site location: inferred from neighbouring sites
*R*[*F*^2^ > 2σ(*F*^2^)] = 0.020	H-atom parameters constrained
*wR*(*F*^2^) = 0.050	*w* = 1/[σ^2^(*F*_o_^2^) + (0.0231*P*)^2^ + 5.9858*P*] where *P* = (*F*_o_^2^ + 2*F*_c_^2^)/3
*S* = 1.05	(Δ/σ)_max_ = 0.002
7152 reflections	Δρ_max_ = 1.10 e Å^−^^3^
370 parameters	Δρ_min_ = −0.65 e Å^−^^3^

### (I) Poly[[tris(nitrato-κ2O,O')cerium(III)]-bis(µ-4,4'-bipyridine N,N'-dioxide-κ2N:N')] Special details

**Table:**

Geometry. All esds (except the esd in the dihedral angle between two l.s. planes) are estimated using the full covariance matrix. The cell esds are taken into account individually in the estimation of esds in distances, angles and torsion angles; correlations between esds in cell parameters are only used when they are defined by crystal symmetry. An approximate (isotropic) treatment of cell esds is used for estimating esds involving l.s. planes.

### (I) Poly[[tris(nitrato-κ2O,O')cerium(III)]-bis(µ-4,4'-bipyridine N,N'-dioxide-κ2N:N')] Fractional atomic coordinates and isotropic or equivalent isotropic displacement parameters (Å^2^)

**Table:**

	*x*	*y*	*z*	*U*_iso_*/*U*_eq_	
Ce1	0.12666 (2)	0.27581 (2)	0.66455 (2)	0.00880 (3)	
O1	0.14943 (4)	1.12935 (8)	0.78743 (9)	0.0138 (2)	
O2	0.07510 (4)	0.41815 (8)	0.70896 (9)	0.0154 (2)	
O3	0.18037 (4)	0.33380 (8)	0.83061 (8)	0.0131 (2)	
O4	0.54328 (4)	0.28358 (8)	1.18738 (9)	0.0126 (2)	
O5	0.19329 (6)	0.41286 (11)	0.65215 (10)	0.0288 (3)	
O6	0.12041 (6)	0.42801 (10)	0.53717 (11)	0.0267 (3)	
O7	0.18842 (7)	0.50937 (10)	0.52255 (12)	0.0365 (4)	
O8	0.21266 (5)	0.18687 (11)	0.66189 (9)	0.0221 (3)	
O9	0.16939 (5)	0.24235 (9)	0.51565 (9)	0.0157 (2)	
O10	0.23607 (5)	0.14464 (9)	0.52773 (9)	0.0195 (2)	
O11	0.05806 (5)	0.25187 (9)	0.48914 (9)	0.0162 (2)	
O12	0.09542 (4)	0.11414 (8)	0.55499 (8)	0.0148 (2)	
O13	0.03572 (5)	0.11024 (10)	0.41152 (9)	0.0217 (3)	
N1	0.14037 (5)	1.03162 (9)	0.77682 (9)	0.0113 (2)	
N2	0.08640 (5)	0.51564 (10)	0.71907 (10)	0.0134 (2)	
N3	0.23131 (5)	0.32751 (10)	0.87085 (9)	0.0109 (2)	
N4	0.49487 (5)	0.28718 (9)	1.12873 (10)	0.0111 (2)	
N5	0.16766 (7)	0.45133 (11)	0.56933 (12)	0.0253 (3)	
N6	0.20687 (5)	0.19053 (10)	0.56676 (10)	0.0145 (2)	
N7	0.06262 (5)	0.15808 (10)	0.48319 (9)	0.0137 (2)	
C1	0.18034 (6)	0.96562 (12)	0.79956 (12)	0.0142 (3)	
H1	0.2150	0.9895	0.8219	0.017*	
C2	0.17085 (6)	0.86374 (12)	0.79041 (12)	0.0144 (3)	
H2	0.1991	0.8180	0.8075	0.017*	
C3	0.12012 (6)	0.82731 (11)	0.75622 (11)	0.0121 (3)	
C4	0.08022 (6)	0.89772 (12)	0.73462 (12)	0.0137 (3)	
H4	0.0452	0.8759	0.7115	0.016*	
C5	0.09092 (6)	0.99873 (12)	0.74644 (12)	0.0136 (3)	
H5	0.0632	1.0457	0.7331	0.016*	
C6	0.05307 (6)	0.58316 (12)	0.66253 (13)	0.0171 (3)	
H6	0.0225	0.5606	0.6144	0.021*	
C7	0.06325 (6)	0.68421 (12)	0.67436 (12)	0.0165 (3)	
H7	0.0393	0.7310	0.6351	0.020*	
C8	0.10854 (6)	0.71893 (11)	0.74362 (12)	0.0124 (3)	
C9	0.14170 (6)	0.64712 (12)	0.80061 (12)	0.0162 (3)	
H9	0.1727	0.6677	0.8485	0.019*	
C10	0.12988 (6)	0.54644 (12)	0.78811 (13)	0.0172 (3)	
H10	0.1525	0.4984	0.8284	0.021*	
C11	0.25885 (6)	0.41124 (11)	0.90585 (11)	0.0128 (3)	
H11	0.2421	0.4747	0.8972	0.015*	
C12	0.31105 (6)	0.40468 (12)	0.95406 (11)	0.0131 (3)	
H12	0.3300	0.4637	0.9793	0.016*	
C13	0.33644 (6)	0.31212 (11)	0.96620 (11)	0.0117 (3)	
C14	0.30687 (6)	0.22856 (11)	0.92576 (12)	0.0137 (3)	
H14	0.3231	0.1647	0.9302	0.016*	
C15	0.25463 (6)	0.23682 (11)	0.87958 (12)	0.0132 (3)	
H15	0.2349	0.1788	0.8538	0.016*	
C16	0.47610 (6)	0.37514 (11)	1.08504 (11)	0.0131 (3)	
H16	0.4985	0.4315	1.0918	0.016*	
C17	0.42495 (6)	0.38381 (12)	1.03088 (12)	0.0135 (3)	
H17	0.4122	0.4460	1.0004	0.016*	
C18	0.39170 (6)	0.30199 (11)	1.02043 (11)	0.0115 (3)	
C19	0.41282 (6)	0.21126 (11)	1.06413 (12)	0.0125 (3)	
H19	0.3915	0.1534	1.0567	0.015*	
C20	0.46424 (6)	0.20521 (12)	1.11776 (12)	0.0129 (3)	
H20	0.4782	0.1433	1.1471	0.015*	

### (I) Poly[[tris(nitrato-κ2O,O')cerium(III)]-bis(µ-4,4'-bipyridine N,N'-dioxide-κ2N:N')] Atomic displacement parameters (Å^2^)

**Table:**

	*U*^11^	*U*^22^	*U*^33^	*U*^12^	*U*^13^	*U*^23^
Ce1	0.00749 (4)	0.00690 (4)	0.01083 (4)	−0.00080 (3)	0.00051 (3)	−0.00044 (2)
O1	0.0156 (5)	0.0062 (5)	0.0175 (5)	−0.0020 (4)	0.0008 (4)	−0.0001 (4)
O2	0.0164 (5)	0.0053 (5)	0.0237 (6)	−0.0017 (4)	0.0043 (4)	−0.0023 (4)
O3	0.0069 (4)	0.0139 (5)	0.0155 (5)	0.0001 (4)	−0.0021 (4)	−0.0013 (4)
O4	0.0063 (5)	0.0135 (5)	0.0158 (5)	0.0010 (4)	−0.0008 (4)	0.0013 (4)
O5	0.0369 (8)	0.0279 (7)	0.0231 (6)	−0.0185 (6)	0.0107 (6)	−0.0048 (5)
O6	0.0349 (7)	0.0180 (6)	0.0309 (7)	0.0042 (5)	0.0152 (6)	0.0061 (5)
O7	0.0665 (11)	0.0128 (6)	0.0464 (9)	−0.0089 (7)	0.0427 (8)	−0.0018 (6)
O8	0.0195 (6)	0.0320 (7)	0.0146 (5)	0.0088 (5)	0.0041 (4)	0.0025 (5)
O9	0.0138 (5)	0.0163 (5)	0.0164 (5)	0.0019 (4)	0.0030 (4)	0.0025 (4)
O10	0.0201 (6)	0.0183 (6)	0.0224 (6)	0.0039 (5)	0.0094 (5)	−0.0015 (5)
O11	0.0169 (5)	0.0131 (5)	0.0170 (5)	0.0016 (4)	0.0018 (4)	0.0001 (4)
O12	0.0154 (5)	0.0117 (5)	0.0151 (5)	0.0000 (4)	0.0006 (4)	−0.0010 (4)
O13	0.0184 (6)	0.0261 (6)	0.0167 (5)	−0.0039 (5)	−0.0016 (4)	−0.0090 (5)
N1	0.0129 (6)	0.0079 (5)	0.0118 (5)	−0.0016 (4)	0.0010 (4)	0.0003 (4)
N2	0.0141 (6)	0.0084 (6)	0.0170 (6)	−0.0003 (5)	0.0033 (5)	−0.0013 (5)
N3	0.0083 (5)	0.0115 (6)	0.0115 (5)	0.0001 (4)	0.0002 (4)	−0.0002 (4)
N4	0.0081 (5)	0.0116 (6)	0.0127 (6)	0.0007 (4)	0.0014 (4)	0.0008 (4)
N5	0.0444 (10)	0.0104 (6)	0.0298 (8)	−0.0066 (6)	0.0244 (7)	−0.0040 (6)
N6	0.0132 (6)	0.0139 (6)	0.0163 (6)	−0.0010 (5)	0.0041 (5)	−0.0004 (5)
N7	0.0119 (6)	0.0169 (6)	0.0117 (5)	−0.0021 (5)	0.0023 (4)	−0.0028 (5)
C1	0.0113 (6)	0.0125 (7)	0.0170 (7)	0.0000 (5)	0.0005 (5)	0.0002 (5)
C2	0.0129 (6)	0.0117 (7)	0.0172 (7)	0.0012 (5)	0.0017 (5)	−0.0002 (5)
C3	0.0147 (7)	0.0083 (6)	0.0117 (6)	−0.0010 (5)	0.0012 (5)	−0.0007 (5)
C4	0.0118 (6)	0.0112 (7)	0.0163 (7)	−0.0011 (5)	0.0010 (5)	0.0003 (5)
C5	0.0113 (6)	0.0112 (7)	0.0163 (7)	0.0002 (5)	0.0005 (5)	0.0007 (5)
C6	0.0155 (7)	0.0120 (7)	0.0196 (7)	−0.0009 (6)	−0.0025 (6)	0.0000 (6)
C7	0.0172 (7)	0.0103 (7)	0.0186 (7)	0.0005 (6)	−0.0010 (6)	0.0018 (6)
C8	0.0143 (7)	0.0084 (6)	0.0139 (7)	−0.0009 (5)	0.0029 (5)	−0.0007 (5)
C9	0.0145 (7)	0.0103 (7)	0.0201 (7)	0.0001 (5)	−0.0015 (6)	−0.0015 (6)
C10	0.0134 (7)	0.0105 (7)	0.0237 (8)	0.0010 (6)	−0.0018 (6)	0.0005 (6)
C11	0.0112 (6)	0.0095 (6)	0.0164 (7)	0.0001 (5)	0.0014 (5)	−0.0014 (5)
C12	0.0099 (6)	0.0111 (6)	0.0163 (7)	−0.0008 (5)	0.0002 (5)	−0.0029 (5)
C13	0.0096 (6)	0.0128 (7)	0.0114 (6)	0.0002 (5)	0.0006 (5)	0.0003 (5)
C14	0.0118 (7)	0.0101 (7)	0.0170 (7)	0.0008 (5)	0.0002 (5)	0.0005 (5)
C15	0.0122 (7)	0.0093 (6)	0.0159 (7)	0.0002 (5)	0.0004 (5)	−0.0004 (5)
C16	0.0108 (6)	0.0112 (6)	0.0158 (7)	−0.0002 (5)	0.0010 (5)	0.0028 (5)
C17	0.0115 (6)	0.0116 (6)	0.0161 (7)	0.0009 (5)	0.0017 (5)	0.0033 (5)
C18	0.0094 (6)	0.0124 (6)	0.0118 (6)	0.0007 (5)	0.0014 (5)	0.0009 (5)
C19	0.0114 (6)	0.0104 (6)	0.0145 (7)	−0.0006 (5)	0.0015 (5)	0.0009 (5)
C20	0.0121 (6)	0.0102 (6)	0.0151 (7)	0.0000 (5)	0.0017 (5)	0.0010 (5)

### (I) Poly[[tris(nitrato-κ2O,O')cerium(III)]-bis(µ-4,4'-bipyridine N,N'-dioxide-κ2N:N')] Geometric parameters (Å, º)

**Table:**

Ce1—O3	2.4685 (11)	C1—H1	0.9500
Ce1—O4^i^	2.4692 (11)	C2—C3	1.398 (2)
Ce1—O2	2.5192 (11)	C2—H2	0.9500
Ce1—O1^ii^	2.5464 (11)	C3—C4	1.393 (2)
Ce1—O5	2.5929 (13)	C3—C8	1.479 (2)
Ce1—O8	2.6004 (12)	C4—C5	1.378 (2)
Ce1—O11	2.6231 (12)	C4—H4	0.9500
Ce1—O12	2.6333 (11)	C5—H5	0.9500
Ce1—O9	2.6428 (12)	C6—C7	1.376 (2)
Ce1—O6	2.6573 (13)	C6—H6	0.9500
O1—N1	1.3268 (16)	C7—C8	1.401 (2)
O1—Ce1^iii^	2.5464 (11)	C7—H7	0.9500
O2—N2	1.3339 (16)	C8—C9	1.393 (2)
O3—N3	1.3277 (15)	C9—C10	1.380 (2)
O4—N4	1.3281 (16)	C9—H9	0.9500
O4—Ce1^iv^	2.4694 (11)	C10—H10	0.9500
O5—N5	1.267 (2)	C11—C12	1.377 (2)
O6—N5	1.260 (2)	C11—H11	0.9500
O7—N5	1.2321 (19)	C12—C13	1.398 (2)
O8—N6	1.2761 (18)	C12—H12	0.9500
O9—N6	1.2629 (18)	C13—C14	1.393 (2)
O10—N6	1.2264 (17)	C13—C18	1.471 (2)
O11—N7	1.2619 (18)	C14—C15	1.374 (2)
O12—N7	1.2718 (17)	C14—H14	0.9500
O13—N7	1.2292 (17)	C15—H15	0.9500
N1—C5	1.3490 (19)	C16—C17	1.374 (2)
N1—C1	1.355 (2)	C16—H16	0.9500
N2—C10	1.350 (2)	C17—C18	1.391 (2)
N2—C6	1.354 (2)	C17—H17	0.9500
N3—C15	1.3517 (19)	C18—C19	1.400 (2)
N3—C11	1.3527 (19)	C19—C20	1.376 (2)
N4—C20	1.3504 (19)	C19—H19	0.9500
N4—C16	1.3512 (19)	C20—H20	0.9500
C1—C2	1.382 (2)		
			
O3—Ce1—O4^i^	107.56 (4)	O7—N5—O5	120.87 (18)
O3—Ce1—O2	76.05 (4)	O6—N5—O5	117.48 (14)
O4^i^—Ce1—O2	68.67 (4)	O10—N6—O9	122.32 (13)
O3—Ce1—O1^ii^	69.67 (4)	O10—N6—O8	121.08 (14)
O4^i^—Ce1—O1^ii^	74.36 (4)	O9—N6—O8	116.59 (13)
O2—Ce1—O1^ii^	117.72 (4)	O13—N7—O11	121.39 (14)
O3—Ce1—O5	66.55 (4)	O13—N7—O12	120.88 (14)
O4^i^—Ce1—O5	153.81 (4)	O11—N7—O12	117.72 (12)
O2—Ce1—O5	85.25 (4)	N1—C1—C2	120.25 (14)
O1^ii^—Ce1—O5	122.70 (4)	N1—C1—H1	119.9
O3—Ce1—O8	82.02 (4)	C2—C1—H1	119.9
O4^i^—Ce1—O8	133.70 (4)	C1—C2—C3	120.67 (14)
O2—Ce1—O8	153.34 (4)	C1—C2—H2	119.7
O1^ii^—Ce1—O8	66.84 (4)	C3—C2—H2	119.7
O5—Ce1—O8	72.09 (5)	C4—C3—C2	117.13 (14)
O3—Ce1—O11	167.04 (4)	C4—C3—C8	120.68 (14)
O4^i^—Ce1—O11	69.34 (4)	C2—C3—C8	122.18 (14)
O2—Ce1—O11	91.27 (4)	C5—C4—C3	120.78 (14)
O1^ii^—Ce1—O11	119.87 (4)	C5—C4—H4	119.6
O5—Ce1—O11	110.32 (4)	C3—C4—H4	119.6
O8—Ce1—O11	109.41 (4)	N1—C5—C4	120.64 (14)
O3—Ce1—O12	143.16 (4)	N1—C5—H5	119.7
O4^i^—Ce1—O12	69.69 (4)	C4—C5—H5	119.7
O2—Ce1—O12	130.28 (4)	N2—C6—C7	120.35 (15)
O1^ii^—Ce1—O12	74.50 (4)	N2—C6—H6	119.8
O5—Ce1—O12	131.05 (4)	C7—C6—H6	119.8
O8—Ce1—O12	76.27 (4)	C6—C7—C8	120.73 (15)
O11—Ce1—O12	48.73 (4)	C6—C7—H7	119.6
O3—Ce1—O9	120.26 (4)	C8—C7—H7	119.6
O4^i^—Ce1—O9	129.97 (4)	C9—C8—C7	117.14 (14)
O2—Ce1—O9	134.21 (4)	C9—C8—C3	121.68 (14)
O1^ii^—Ce1—O9	108.00 (4)	C7—C8—C3	121.18 (14)
O5—Ce1—O9	67.46 (4)	C10—C9—C8	120.65 (15)
O8—Ce1—O9	48.65 (4)	C10—C9—H9	119.7
O11—Ce1—O9	67.00 (4)	C8—C9—H9	119.7
O12—Ce1—O9	63.59 (4)	N2—C10—C9	120.55 (14)
O3—Ce1—O6	106.67 (4)	N2—C10—H10	119.7
O4^i^—Ce1—O6	115.64 (4)	C9—C10—H10	119.7
O2—Ce1—O6	69.15 (4)	N3—C11—C12	120.16 (14)
O1^ii^—Ce1—O6	169.94 (4)	N3—C11—H11	119.9
O5—Ce1—O6	48.58 (5)	C12—C11—H11	119.9
O8—Ce1—O6	103.65 (4)	C11—C12—C13	120.71 (14)
O11—Ce1—O6	65.43 (4)	C11—C12—H12	119.6
O12—Ce1—O6	107.17 (4)	C13—C12—H12	119.6
O9—Ce1—O6	65.20 (4)	C14—C13—C12	117.02 (14)
N1—O1—Ce1^iii^	132.64 (9)	C14—C13—C18	120.87 (14)
N2—O2—Ce1	129.52 (9)	C12—C13—C18	122.11 (14)
N3—O3—Ce1	129.72 (9)	C15—C14—C13	121.13 (14)
N4—O4—Ce1^iv^	134.54 (9)	C15—C14—H14	119.4
N5—O5—Ce1	97.52 (10)	C13—C14—H14	119.4
N5—O6—Ce1	94.62 (10)	N3—C15—C14	120.03 (14)
N6—O8—Ce1	97.86 (9)	N3—C15—H15	120.0
N6—O9—Ce1	96.18 (9)	C14—C15—H15	120.0
N7—O11—Ce1	97.15 (8)	N4—C16—C17	120.61 (14)
N7—O12—Ce1	96.39 (8)	N4—C16—H16	119.7
O1—N1—C5	119.22 (13)	C17—C16—H16	119.7
O1—N1—C1	120.25 (13)	C16—C17—C18	120.36 (14)
C5—N1—C1	120.49 (13)	C16—C17—H17	119.8
O2—N2—C10	119.84 (13)	C18—C17—H17	119.8
O2—N2—C6	119.56 (13)	C17—C18—C19	117.49 (14)
C10—N2—C6	120.56 (14)	C17—C18—C13	120.50 (14)
O3—N3—C15	119.41 (13)	C19—C18—C13	122.01 (14)
O3—N3—C11	119.65 (12)	C20—C19—C18	120.59 (14)
C15—N3—C11	120.89 (13)	C20—C19—H19	119.7
O4—N4—C20	120.08 (12)	C18—C19—H19	119.7
O4—N4—C16	118.99 (12)	N4—C20—C19	120.07 (14)
C20—N4—C16	120.83 (13)	N4—C20—H20	120.0
O7—N5—O6	121.65 (18)	C19—C20—H20	120.0
			
Ce1—O3—O4—Ce1^iv^	5.38 (7)	Ce1—O2—O1—Ce1^iii^	92.53 (6)

Symmetry codes: (i) *x*−1/2, −*y*+1/2, *z*−1/2; (ii) *x*, *y*−1, *z*; (iii) *x*, *y*+1, *z*; (iv) *x*+1/2, −*y*+1/2, *z*+1/2.

### (I) Poly[[tris(nitrato-κ2O,O')cerium(III)]-bis(µ-4,4'-bipyridine N,N'-dioxide-κ2N:N')] Hydrogen-bond geometry (Å, º)

**Table:**

*D*—H···*A*	*D*—H	H···*A*	*D*···*A*	*D*—H···*A*
C1—H1···O5^v^	0.95	2.59	3.342 (2)	136
C4—H4···O13^vi^	0.95	2.37	3.208 (2)	148
C5—H5···O4^vii^	0.95	2.38	3.1868 (19)	142
C9—H9···O9^viii^	0.95	2.62	3.206 (2)	121
C9—H9···O10^v^	0.95	2.59	3.475 (2)	156
C10—H10···O3	0.95	2.32	3.128 (2)	143
C10—H10···O7^viii^	0.95	2.58	3.264 (2)	129
C11—H11···O10^v^	0.95	2.49	3.237 (2)	135
C14—H14···O7^ix^	0.95	2.22	3.004 (2)	139
C15—H15···O1^ii^	0.95	2.32	3.1069 (19)	140
C16—H16···O13^v^	0.95	2.55	3.154 (2)	122
C17—H17···O12^v^	0.95	2.36	3.2837 (19)	164
C20—H20···O2^iv^	0.95	2.63	3.3265 (19)	130

Symmetry codes: (ii) *x*, *y*−1, *z*; (iv) *x*+1/2, −*y*+1/2, *z*+1/2; (v) −*x*+1/2, *y*+1/2, −*z*+3/2; (vi) −*x*, −*y*+1, −*z*+1; (vii) *x*−1/2, −*y*+3/2, *z*−1/2; (viii) *x*, −*y*+1, *z*+1/2; (ix) −*x*+1/2, *y*−1/2, −*z*+3/2.

### (II) Poly[[tris(nitrato-κ2O,O')praeseodymium(III)]-bis(µ-4,4'-bipyridine N,N'-dioxide-κ2N:N')] Crystal data

**Table:**

[Pr(NO_3_)_3_(C_10_H_8_N_2_O_2_)_2_]	*F*(000) = 2784
*M**_r_* = 703.31	*D*_x_ = 1.984 Mg m^−^^3^
Monoclinic, *C*2/*c*	Mo *K*α radiation, λ = 0.71073 Å
Hall symbol: -C 2yc	Cell parameters from 11450 reflections
*a* = 26.7416 (18) Å	θ = 2.5–31.3°
*b* = 13.3127 (9) Å	µ = 2.16 mm^−^^1^
*c* = 13.7586 (9) Å	*T* = 173 K
β = 105.981 (1)°	Block, yellow
*V* = 4708.8 (5) Å^3^	0.55 × 0.37 × 0.26 mm
*Z* = 8	

### (II) Poly[[tris(nitrato-κ2O,O')praeseodymium(III)]-bis(µ-4,4'-bipyridine N,N'-dioxide-κ2N:N')] Data collection

**Table:**

Bruker APEXII CCD diffractometer	7241 independent reflections
Radiation source: sealed tube	6782 reflections with *I* > 2σ(*I*)
Graphite monochromator	*R*_int_ = 0.020
phi and ω scans	θ_max_ = 31.6°, θ_min_ = 1.6°
Absorption correction: multi-scan (SADABS; Bruker, 2009)	*h* = −39→38
*T*_min_ = 0.579, *T*_max_ = 0.746	*k* = −18→18
18363 measured reflections	*l* = −19→15

### (II) Poly[[tris(nitrato-κ2O,O')praeseodymium(III)]-bis(µ-4,4'-bipyridine N,N'-dioxide-κ2N:N')] Refinement

**Table:**

Refinement on *F*^2^	0 restraints
Least-squares matrix: full	Hydrogen site location: inferred from neighbouring sites
*R*[*F*^2^ > 2σ(*F*^2^)] = 0.021	H-atom parameters constrained
*wR*(*F*^2^) = 0.052	*w* = 1/[σ^2^(*F*_o_^2^) + (0.0248*P*)^2^ + 6.7123*P*] where *P* = (*F*_o_^2^ + 2*F*_c_^2^)/3
*S* = 1.05	(Δ/σ)_max_ = 0.003
7241 reflections	Δρ_max_ = 0.89 e Å^−^^3^
370 parameters	Δρ_min_ = −1.06 e Å^−^^3^

### (II) Poly[[tris(nitrato-κ2O,O')praeseodymium(III)]-bis(µ-4,4'-bipyridine N,N'-dioxide-κ2N:N')] Special details

**Table:**

Geometry. All esds (except the esd in the dihedral angle between two l.s. planes) are estimated using the full covariance matrix. The cell esds are taken into account individually in the estimation of esds in distances, angles and torsion angles; correlations between esds in cell parameters are only used when they are defined by crystal symmetry. An approximate (isotropic) treatment of cell esds is used for estimating esds involving l.s. planes.

### (II) Poly[[tris(nitrato-κ2O,O')praeseodymium(III)]-bis(µ-4,4'-bipyridine N,N'-dioxide-κ2N:N')] Fractional atomic coordinates and isotropic or equivalent isotropic displacement parameters (Å^2^)

**Table:**

	*x*	*y*	*z*	*U*_iso_*/*U*_eq_	
Pr1	0.12674 (2)	0.27553 (2)	0.66470 (2)	0.00790 (3)	
O1	0.14910 (4)	1.12982 (8)	0.78780 (9)	0.0121 (2)	
O2	0.07540 (5)	0.41686 (8)	0.70889 (10)	0.0138 (2)	
O3	0.17985 (4)	0.33321 (9)	0.83058 (9)	0.0115 (2)	
O4	0.54388 (4)	0.28390 (8)	1.18793 (9)	0.0111 (2)	
O5	0.19376 (6)	0.41071 (11)	0.65327 (11)	0.0253 (3)	
O6	0.12068 (6)	0.42773 (10)	0.53849 (11)	0.0236 (3)	
O7	0.18928 (7)	0.50822 (10)	0.52428 (13)	0.0324 (4)	
O8	0.21197 (5)	0.18554 (11)	0.66227 (10)	0.0196 (3)	
O9	0.16890 (5)	0.24298 (10)	0.51644 (10)	0.0144 (2)	
O10	0.23564 (5)	0.14508 (10)	0.52791 (10)	0.0181 (2)	
O11	0.05832 (5)	0.25265 (10)	0.49034 (9)	0.0144 (2)	
O12	0.09581 (4)	0.11451 (9)	0.55595 (9)	0.0135 (2)	
O13	0.03605 (5)	0.11082 (10)	0.41182 (10)	0.0198 (3)	
N1	0.14024 (5)	1.03189 (10)	0.77697 (10)	0.0100 (2)	
N2	0.08667 (5)	0.51456 (10)	0.71901 (11)	0.0117 (2)	
N3	0.23089 (5)	0.32704 (10)	0.87050 (10)	0.0096 (2)	
N4	0.49530 (5)	0.28712 (10)	1.12884 (11)	0.0097 (2)	
N5	0.16820 (7)	0.45013 (11)	0.57063 (13)	0.0219 (3)	
N6	0.20645 (5)	0.19037 (11)	0.56710 (11)	0.0129 (3)	
N7	0.06291 (5)	0.15870 (11)	0.48410 (10)	0.0124 (3)	
C1	0.18032 (6)	0.96583 (12)	0.79944 (13)	0.0127 (3)	
H1	0.2151	0.9899	0.8217	0.015*	
C2	0.17102 (6)	0.86383 (12)	0.79021 (13)	0.0130 (3)	
H2	0.1994	0.8182	0.8072	0.016*	
C3	0.12013 (6)	0.82702 (12)	0.75610 (12)	0.0106 (3)	
C4	0.07981 (6)	0.89758 (12)	0.73481 (13)	0.0126 (3)	
H4	0.0447	0.8756	0.7120	0.015*	
C5	0.09060 (6)	0.99871 (12)	0.74667 (12)	0.0120 (3)	
H5	0.0628	1.0457	0.7334	0.014*	
C6	0.05349 (6)	0.58208 (12)	0.66223 (13)	0.0152 (3)	
H6	0.0230	0.5593	0.6138	0.018*	
C7	0.06337 (6)	0.68346 (12)	0.67370 (13)	0.0145 (3)	
H7	0.0394	0.7301	0.6340	0.017*	
C8	0.10860 (6)	0.71840 (11)	0.74358 (13)	0.0109 (3)	
C9	0.14177 (6)	0.64669 (12)	0.80127 (13)	0.0149 (3)	
H9	0.1727	0.6674	0.8499	0.018*	
C10	0.12999 (6)	0.54590 (12)	0.78830 (14)	0.0154 (3)	
H10	0.1527	0.4979	0.8288	0.019*	
C11	0.25850 (6)	0.41103 (12)	0.90557 (12)	0.0116 (3)	
H11	0.2417	0.4746	0.8969	0.014*	
C12	0.31102 (6)	0.40482 (12)	0.95382 (13)	0.0120 (3)	
H12	0.3300	0.4640	0.9790	0.014*	
C13	0.33636 (6)	0.31196 (12)	0.96590 (12)	0.0100 (3)	
C14	0.30678 (6)	0.22808 (11)	0.92570 (13)	0.0122 (3)	
H14	0.3231	0.1642	0.9302	0.015*	
C15	0.25433 (6)	0.23623 (12)	0.87957 (13)	0.0116 (3)	
H15	0.2346	0.1780	0.8540	0.014*	
C16	0.47654 (6)	0.37537 (12)	1.08487 (12)	0.0117 (3)	
H16	0.4990	0.4318	1.0914	0.014*	
C17	0.42522 (6)	0.38374 (12)	1.03083 (13)	0.0120 (3)	
H17	0.4124	0.4461	1.0005	0.014*	
C18	0.39185 (6)	0.30165 (12)	1.02012 (12)	0.0102 (3)	
C19	0.41298 (6)	0.21084 (12)	1.06397 (13)	0.0113 (3)	
H19	0.3916	0.1528	1.0566	0.014*	
C20	0.46461 (6)	0.20508 (12)	1.11778 (13)	0.0116 (3)	
H20	0.4787	0.1431	1.1472	0.014*	

### (II) Poly[[tris(nitrato-κ2O,O')praeseodymium(III)]-bis(µ-4,4'-bipyridine N,N'-dioxide-κ2N:N')] Atomic displacement parameters (Å^2^)

**Table:**

	*U*^11^	*U*^22^	*U*^33^	*U*^12^	*U*^13^	*U*^23^
Pr1	0.00728 (4)	0.00661 (4)	0.00934 (5)	−0.00065 (2)	0.00148 (3)	−0.00049 (3)
O1	0.0148 (5)	0.0056 (5)	0.0146 (5)	−0.0022 (4)	0.0018 (4)	−0.0004 (4)
O2	0.0154 (5)	0.0052 (5)	0.0209 (6)	−0.0015 (4)	0.0053 (5)	−0.0021 (4)
O3	0.0063 (4)	0.0124 (5)	0.0137 (5)	0.0004 (4)	−0.0007 (4)	−0.0013 (4)
O4	0.0065 (5)	0.0123 (5)	0.0129 (5)	0.0013 (4)	0.0001 (4)	0.0010 (4)
O5	0.0328 (7)	0.0242 (7)	0.0209 (7)	−0.0155 (6)	0.0106 (6)	−0.0031 (6)
O6	0.0300 (7)	0.0164 (6)	0.0278 (7)	0.0033 (5)	0.0139 (6)	0.0046 (5)
O7	0.0589 (10)	0.0118 (6)	0.0418 (9)	−0.0076 (6)	0.0397 (8)	−0.0016 (6)
O8	0.0178 (6)	0.0292 (7)	0.0122 (6)	0.0078 (5)	0.0046 (5)	0.0029 (5)
O9	0.0129 (5)	0.0150 (5)	0.0146 (6)	0.0029 (4)	0.0029 (4)	0.0024 (5)
O10	0.0188 (6)	0.0181 (6)	0.0205 (6)	0.0040 (5)	0.0105 (5)	−0.0012 (5)
O11	0.0149 (5)	0.0119 (5)	0.0152 (6)	0.0010 (4)	0.0020 (5)	−0.0003 (5)
O12	0.0145 (5)	0.0112 (5)	0.0130 (5)	0.0008 (4)	0.0009 (4)	−0.0001 (4)
O13	0.0181 (6)	0.0232 (7)	0.0154 (6)	−0.0038 (5)	0.0000 (5)	−0.0078 (5)
N1	0.0129 (6)	0.0075 (6)	0.0094 (6)	−0.0014 (4)	0.0024 (5)	0.0000 (5)
N2	0.0128 (6)	0.0068 (6)	0.0157 (6)	−0.0005 (4)	0.0043 (5)	−0.0013 (5)
N3	0.0079 (5)	0.0102 (6)	0.0102 (6)	0.0001 (4)	0.0016 (4)	0.0000 (5)
N4	0.0077 (5)	0.0109 (6)	0.0106 (6)	0.0006 (4)	0.0028 (5)	0.0003 (5)
N5	0.0391 (9)	0.0081 (6)	0.0260 (8)	−0.0043 (6)	0.0216 (7)	−0.0035 (6)
N6	0.0121 (6)	0.0125 (6)	0.0148 (7)	0.0002 (5)	0.0048 (5)	0.0000 (5)
N7	0.0112 (6)	0.0152 (6)	0.0108 (6)	−0.0018 (5)	0.0031 (5)	−0.0029 (5)
C1	0.0107 (6)	0.0121 (7)	0.0146 (7)	0.0003 (5)	0.0022 (6)	−0.0003 (6)
C2	0.0119 (7)	0.0112 (7)	0.0146 (7)	0.0012 (5)	0.0017 (6)	−0.0009 (6)
C3	0.0137 (7)	0.0072 (6)	0.0107 (7)	−0.0007 (5)	0.0031 (5)	0.0000 (5)
C4	0.0113 (6)	0.0105 (7)	0.0149 (7)	−0.0007 (5)	0.0018 (6)	0.0007 (6)
C5	0.0120 (6)	0.0094 (7)	0.0137 (7)	0.0001 (5)	0.0021 (6)	0.0006 (6)
C6	0.0141 (7)	0.0111 (7)	0.0176 (8)	−0.0016 (5)	−0.0005 (6)	0.0007 (6)
C7	0.0157 (7)	0.0101 (7)	0.0152 (8)	−0.0006 (5)	−0.0001 (6)	0.0009 (6)
C8	0.0132 (7)	0.0077 (7)	0.0120 (7)	−0.0002 (5)	0.0037 (6)	−0.0009 (5)
C9	0.0135 (7)	0.0100 (7)	0.0186 (8)	0.0001 (5)	−0.0001 (6)	−0.0010 (6)
C10	0.0132 (7)	0.0095 (7)	0.0205 (8)	0.0013 (5)	−0.0006 (6)	0.0006 (6)
C11	0.0111 (6)	0.0084 (6)	0.0143 (7)	0.0001 (5)	0.0018 (6)	−0.0010 (6)
C12	0.0100 (6)	0.0097 (7)	0.0152 (7)	−0.0013 (5)	0.0019 (5)	−0.0015 (6)
C13	0.0086 (6)	0.0111 (7)	0.0100 (7)	−0.0006 (5)	0.0019 (5)	0.0004 (5)
C14	0.0113 (7)	0.0088 (7)	0.0151 (7)	0.0003 (5)	0.0015 (6)	0.0003 (6)
C15	0.0113 (7)	0.0082 (6)	0.0141 (7)	0.0000 (5)	0.0011 (6)	−0.0001 (6)
C16	0.0100 (6)	0.0099 (7)	0.0148 (7)	−0.0002 (5)	0.0027 (5)	0.0027 (6)
C17	0.0102 (6)	0.0097 (7)	0.0153 (7)	0.0004 (5)	0.0023 (6)	0.0027 (6)
C18	0.0084 (6)	0.0112 (7)	0.0106 (7)	−0.0001 (5)	0.0020 (5)	0.0000 (6)
C19	0.0107 (6)	0.0103 (7)	0.0127 (7)	−0.0008 (5)	0.0029 (5)	0.0003 (6)
C20	0.0113 (6)	0.0101 (7)	0.0131 (7)	0.0005 (5)	0.0029 (6)	0.0011 (6)

### (II) Poly[[tris(nitrato-κ2O,O')praeseodymium(III)]-bis(µ-4,4'-bipyridine N,N'-dioxide-κ2N:N')] Geometric parameters (Å, º)

**Table:**

Pr1—O4^i^	2.4554 (12)	C1—H1	0.9500
Pr1—O3	2.4558 (11)	C2—C3	1.400 (2)
Pr1—O2	2.5009 (12)	C2—H2	0.9500
Pr1—O1^ii^	2.5360 (12)	C3—C4	1.399 (2)
Pr1—O5	2.5750 (13)	C3—C8	1.479 (2)
Pr1—O8	2.5832 (13)	C4—C5	1.377 (2)
Pr1—O11	2.6036 (12)	C4—H4	0.9500
Pr1—O12	2.6147 (12)	C5—H5	0.9500
Pr1—O9	2.6242 (13)	C6—C7	1.376 (2)
Pr1—O6	2.6443 (14)	C6—H6	0.9500
O1—N1	1.3261 (17)	C7—C8	1.401 (2)
O1—Pr1^iii^	2.5360 (12)	C7—H7	0.9500
O2—N2	1.3337 (17)	C8—C9	1.393 (2)
O3—N3	1.3261 (16)	C9—C10	1.379 (2)
O4—N4	1.3300 (17)	C9—H9	0.9500
O4—Pr1^iv^	2.4554 (12)	C10—H10	0.9500
O5—N5	1.268 (2)	C11—C12	1.381 (2)
O6—N5	1.261 (2)	C11—H11	0.9500
O7—N5	1.233 (2)	C12—C13	1.397 (2)
O8—N6	1.2782 (19)	C12—H12	0.9500
O9—N6	1.2646 (18)	C13—C14	1.392 (2)
O10—N6	1.2229 (18)	C13—C18	1.472 (2)
O11—N7	1.2620 (18)	C14—C15	1.375 (2)
O12—N7	1.2722 (18)	C14—H14	0.9500
O13—N7	1.2319 (18)	C15—H15	0.9500
N1—C5	1.352 (2)	C16—C17	1.373 (2)
N1—C1	1.355 (2)	C16—H16	0.9500
N2—C10	1.348 (2)	C17—C18	1.393 (2)
N2—C6	1.351 (2)	C17—H17	0.9500
N3—C15	1.352 (2)	C18—C19	1.399 (2)
N3—C11	1.353 (2)	C19—C20	1.377 (2)
N4—C20	1.349 (2)	C19—H19	0.9500
N4—C16	1.353 (2)	C20—H20	0.9500
C1—C2	1.380 (2)		
			
O4^i^—Pr1—O3	106.95 (4)	O4—N4—C16	118.73 (13)
O4^i^—Pr1—O2	68.60 (4)	C20—N4—C16	120.89 (14)
O3—Pr1—O2	75.79 (4)	O7—N5—O6	121.81 (18)
O4^i^—Pr1—O1^ii^	73.85 (4)	O7—N5—O5	120.86 (18)
O3—Pr1—O1^ii^	69.44 (4)	O6—N5—O5	117.33 (15)
O2—Pr1—O1^ii^	117.22 (4)	O7—N5—Pr1	168.42 (12)
O4^i^—Pr1—O5	154.35 (4)	O6—N5—Pr1	60.94 (9)
O3—Pr1—O5	66.68 (4)	O5—N5—Pr1	57.81 (8)
O2—Pr1—O5	85.82 (5)	O10—N6—O9	122.44 (15)
O1^ii^—Pr1—O5	122.36 (4)	O10—N6—O8	121.27 (14)
O4^i^—Pr1—O8	133.11 (4)	O9—N6—O8	116.28 (14)
O3—Pr1—O8	82.56 (4)	O13—N7—O11	121.44 (14)
O2—Pr1—O8	153.82 (4)	O13—N7—O12	120.85 (14)
O1^ii^—Pr1—O8	66.88 (4)	O11—N7—O12	117.70 (13)
O5—Pr1—O8	72.05 (5)	N1—C1—C2	120.45 (14)
O4^i^—Pr1—O11	69.62 (4)	N1—C1—H1	119.8
O3—Pr1—O11	166.61 (4)	C2—C1—H1	119.8
O2—Pr1—O11	91.05 (4)	C1—C2—C3	120.60 (15)
O1^ii^—Pr1—O11	120.17 (4)	C1—C2—H2	119.7
O5—Pr1—O11	110.48 (4)	C3—C2—H2	119.7
O8—Pr1—O11	109.43 (4)	C4—C3—C2	117.20 (14)
O4^i^—Pr1—O12	69.86 (4)	C4—C3—C8	120.55 (14)
O3—Pr1—O12	143.04 (4)	C2—C3—C8	122.25 (14)
O2—Pr1—O12	130.35 (4)	C5—C4—C3	120.50 (14)
O1^ii^—Pr1—O12	74.59 (4)	C5—C4—H4	119.8
O5—Pr1—O12	130.84 (4)	C3—C4—H4	119.8
O8—Pr1—O12	75.74 (4)	N1—C5—C4	120.79 (15)
O11—Pr1—O12	49.12 (4)	N1—C5—H5	119.6
O4^i^—Pr1—O9	130.11 (4)	C4—C5—H5	119.6
O3—Pr1—O9	120.78 (4)	N2—C6—C7	120.78 (15)
O2—Pr1—O9	134.05 (4)	N2—C6—H6	119.6
O1^ii^—Pr1—O9	108.67 (4)	C7—C6—H6	119.6
O5—Pr1—O9	67.21 (4)	C6—C7—C8	120.43 (15)
O8—Pr1—O9	49.00 (4)	C6—C7—H7	119.8
O11—Pr1—O9	66.91 (4)	C8—C7—H7	119.8
O12—Pr1—O9	63.63 (4)	C9—C8—C7	117.22 (14)
O4^i^—Pr1—O6	115.98 (4)	C9—C8—C3	121.62 (14)
O3—Pr1—O6	106.61 (4)	C7—C8—C3	121.16 (14)
O2—Pr1—O6	69.19 (4)	C10—C9—C8	120.43 (15)
O1^ii^—Pr1—O6	170.15 (4)	C10—C9—H9	119.8
O5—Pr1—O6	48.87 (5)	C8—C9—H9	119.8
O8—Pr1—O6	103.98 (4)	N2—C10—C9	120.93 (15)
O11—Pr1—O6	65.42 (4)	N2—C10—H10	119.5
O12—Pr1—O6	107.45 (4)	C9—C10—H10	119.5
O9—Pr1—O6	65.03 (4)	N3—C11—C12	120.32 (14)
O4^i^—Pr1—N5	139.09 (4)	N3—C11—H11	119.8
O3—Pr1—N5	88.34 (4)	C12—C11—H11	119.8
O2—Pr1—N5	79.39 (4)	C11—C12—C13	120.37 (14)
O1^ii^—Pr1—N5	146.01 (4)	C11—C12—H12	119.8
O5—Pr1—N5	24.63 (5)	C13—C12—H12	119.8
O8—Pr1—N5	85.53 (5)	C14—C13—C12	117.30 (14)
O11—Pr1—N5	86.73 (5)	C14—C13—C18	120.64 (14)
O12—Pr1—N5	118.82 (4)	C12—C13—C18	122.05 (14)
O9—Pr1—N5	60.42 (4)	C15—C14—C13	121.05 (15)
O6—Pr1—N5	24.63 (5)	C15—C14—H14	119.5
N1—O1—Pr1^iii^	132.45 (9)	C13—C14—H14	119.5
N2—O2—Pr1	129.47 (9)	N3—C15—C14	120.09 (14)
N3—O3—Pr1	129.16 (9)	N3—C15—H15	120.0
N4—O4—Pr1^iv^	134.06 (10)	C14—C15—H15	120.0
N5—O5—Pr1	97.56 (10)	N4—C16—C17	120.31 (14)
N5—O6—Pr1	94.43 (10)	N4—C16—H16	119.8
N6—O8—Pr1	97.84 (9)	C17—C16—H16	119.8
N6—O9—Pr1	96.25 (9)	C16—C17—C18	120.61 (15)
N7—O11—Pr1	97.00 (9)	C16—C17—H17	119.7
N7—O12—Pr1	96.18 (9)	C18—C17—H17	119.7
O1—N1—C5	119.07 (13)	C17—C18—C19	117.47 (14)
O1—N1—C1	120.46 (13)	C17—C18—C13	120.49 (14)
C5—N1—C1	120.42 (14)	C19—C18—C13	122.02 (14)
O2—N2—C10	120.08 (13)	C20—C19—C18	120.40 (15)
O2—N2—C6	119.67 (13)	C20—C19—H19	119.8
C10—N2—C6	120.20 (14)	C18—C19—H19	119.8
O3—N3—C15	119.46 (13)	N4—C20—C19	120.27 (15)
O3—N3—C11	119.65 (13)	N4—C20—H20	119.9
C15—N3—C11	120.82 (13)	C19—C20—H20	119.9
O4—N4—C20	120.28 (13)		

Symmetry codes: (i) *x*−1/2, −*y*+1/2, *z*−1/2; (ii) *x*, *y*−1, *z*; (iii) *x*, *y*+1, *z*; (iv) *x*+1/2, −*y*+1/2, *z*+1/2.

### (II) Poly[[tris(nitrato-κ2O,O')praeseodymium(III)]-bis(µ-4,4'-bipyridine N,N'-dioxide-κ2N:N')] Hydrogen-bond geometry (Å, º)

**Table:**

*D*—H···*A*	*D*—H	H···*A*	*D*···*A*	*D*—H···*A*
C1—H1···O5^v^	0.95	2.59	3.331 (2)	135
C4—H4···O13^vi^	0.95	2.36	3.200 (2)	147
C5—H5···O4^vii^	0.95	2.37	3.168 (2)	141
C9—H9···O9^viii^	0.95	2.61	3.204 (2)	121
C9—H9···O10^v^	0.95	2.58	3.468 (2)	156
C10—H10···O3	0.95	2.31	3.115 (2)	143
C10—H10···O7^viii^	0.95	2.60	3.277 (3)	129
C11—H11···O10^v^	0.95	2.50	3.239 (2)	135
C14—H14···O7^ix^	0.95	2.22	3.002 (2)	139
C15—H15···O1^ii^	0.95	2.31	3.0924 (19)	140
C16—H16···O13^v^	0.95	2.56	3.154 (2)	121
C17—H17···O12^v^	0.95	2.36	3.288 (2)	164
C20—H20···O2^iv^	0.95	2.62	3.307 (2)	130

Symmetry codes: (ii) *x*, *y*−1, *z*; (iv) *x*+1/2, −*y*+1/2, *z*+1/2; (v) −*x*+1/2, *y*+1/2, −*z*+3/2; (vi) −*x*, −*y*+1, −*z*+1; (vii) *x*−1/2, −*y*+3/2, *z*−1/2; (viii) *x*, −*y*+1, *z*+1/2; (ix) −*x*+1/2, *y*−1/2, −*z*+3/2.

### (III) Poly[[tris(nitrato-κ2O,O')neodymium(III)]-bis(µ-4,4'-bipyridine N,N'-dioxide-κ2N:N'}] Crystal data

**Table:**

[Nd(NO_3_)_3_(C_10_H_8_N_2_O_2_)_2_]	*F*(000) = 2792
*M**_r_* = 706.64	*D*_x_ = 1.993 Mg m^−^^3^
Monoclinic, *C*2/*c*	Mo *K*α radiation, λ = 0.71073 Å
*a* = 26.7422 (10) Å	Cell parameters from 8852 reflections
*b* = 13.3035 (5) Å	θ = 2.5–31.4°
*c* = 13.7804 (5) Å	µ = 2.29 mm^−^^1^
β = 106.065 (1)°	*T* = 173 K
*V* = 4711.1 (3) Å^3^	Block, yellow
*Z* = 8	0.14 × 0.12 × 0.08 mm

### (III) Poly[[tris(nitrato-κ2O,O')neodymium(III)]-bis(µ-4,4'-bipyridine N,N'-dioxide-κ2N:N'}] Data collection

**Table:**

Bruker D8 Quest CMOS diffractometer	8277 independent reflections
Radiation source: I-mu-S microsource X-ray tube	5419 reflections with *I* > 2σ(*I*)
Laterally graded multilayer (Goebel) mirror monochromator	*R*_int_ = 0.115
ω and phi scans	θ_max_ = 33.5°, θ_min_ = 2.2°
Absorption correction: multi-scan (SADABS; Bruker, 2009)	*h* = −38→39
*T*_min_ = 0.682, *T*_max_ = 0.747	*k* = −20→18
47148 measured reflections	*l* = −21→18

### (III) Poly[[tris(nitrato-κ2O,O')neodymium(III)]-bis(µ-4,4'-bipyridine N,N'-dioxide-κ2N:N'}] Refinement

**Table:**

Refinement on *F*^2^	0 restraints
Least-squares matrix: full	Hydrogen site location: inferred from neighbouring sites
*R*[*F*^2^ > 2σ(*F*^2^)] = 0.051	H-atom parameters constrained
*wR*(*F*^2^) = 0.067	*w* = 1/[σ^2^(*F*_o_^2^) + (0.0161*P*)^2^ + 13.5513*P*] where *P* = (*F*_o_^2^ + 2*F*_c_^2^)/3
*S* = 1.01	(Δ/σ)_max_ = 0.001
8277 reflections	Δρ_max_ = 1.49 e Å^−^^3^
370 parameters	Δρ_min_ = −1.29 e Å^−^^3^

### (III) Poly[[tris(nitrato-κ2O,O')neodymium(III)]-bis(µ-4,4'-bipyridine N,N'-dioxide-κ2N:N'}] Special details

**Table:**

Geometry. All esds (except the esd in the dihedral angle between two l.s. planes) are estimated using the full covariance matrix. The cell esds are taken into account individually in the estimation of esds in distances, angles and torsion angles; correlations between esds in cell parameters are only used when they are defined by crystal symmetry. An approximate (isotropic) treatment of cell esds is used for estimating esds involving l.s. planes.

### (III) Poly[[tris(nitrato-κ2O,O')neodymium(III)]-bis(µ-4,4'-bipyridine N,N'-dioxide-κ2N:N'}] Fractional atomic coordinates and isotropic or equivalent isotropic displacement parameters (Å^2^)

**Table:**

	*x*	*y*	*z*	*U*_iso_*/*U*_eq_	
Nd1	0.12681 (2)	0.27502 (2)	0.66431 (2)	0.00728 (4)	
O1	0.14904 (8)	1.13013 (15)	0.78718 (16)	0.0116 (5)	
O2	0.07574 (8)	0.41599 (15)	0.70766 (16)	0.0130 (5)	
O3	0.17968 (8)	0.33233 (15)	0.83003 (15)	0.0111 (4)	
O4	0.54422 (8)	0.28373 (16)	1.18789 (15)	0.0104 (4)	
O5	0.19319 (10)	0.40971 (18)	0.65362 (18)	0.0251 (6)	
O6	0.12057 (10)	0.42656 (17)	0.53782 (19)	0.0236 (6)	
O7	0.18928 (11)	0.50787 (17)	0.5251 (2)	0.0317 (7)	
O8	0.21193 (9)	0.18587 (18)	0.66255 (16)	0.0190 (5)	
O9	0.16865 (8)	0.24311 (15)	0.51655 (16)	0.0139 (5)	
O10	0.23559 (9)	0.14500 (17)	0.52853 (17)	0.0187 (5)	
O11	0.05854 (8)	0.25293 (15)	0.49154 (16)	0.0136 (5)	
O12	0.09612 (8)	0.11469 (16)	0.55686 (16)	0.0120 (5)	
O13	0.03619 (9)	0.11085 (17)	0.41220 (17)	0.0198 (5)	
N1	0.14002 (10)	1.03179 (18)	0.77624 (18)	0.0092 (5)	
N2	0.08697 (10)	0.51388 (19)	0.7183 (2)	0.0113 (6)	
N3	0.23077 (10)	0.32610 (19)	0.87052 (18)	0.0094 (5)	
N4	0.49563 (9)	0.28710 (19)	1.12926 (17)	0.0094 (5)	
N5	0.16807 (13)	0.4492 (2)	0.5712 (2)	0.0228 (7)	
N6	0.20618 (10)	0.19050 (19)	0.56738 (19)	0.0125 (6)	
N7	0.06299 (10)	0.1590 (2)	0.48487 (19)	0.0124 (6)	
C1	0.18004 (12)	0.9658 (2)	0.7982 (2)	0.0123 (6)	
H1	0.2148	0.9899	0.8201	0.015*	
C2	0.17080 (12)	0.8640 (2)	0.7892 (2)	0.0120 (6)	
H2	0.1993	0.8185	0.8059	0.014*	
C3	0.12022 (12)	0.8270 (2)	0.7557 (2)	0.0097 (6)	
C4	0.07995 (12)	0.8974 (2)	0.7348 (2)	0.0117 (6)	
H4	0.0449	0.8751	0.7123	0.014*	
C5	0.09037 (12)	0.9987 (2)	0.7464 (2)	0.0121 (6)	
H5	0.0625	1.0455	0.7334	0.015*	
C6	0.05363 (13)	0.5813 (2)	0.6616 (2)	0.0144 (7)	
H6	0.0230	0.5584	0.6133	0.017*	
C7	0.06354 (12)	0.6827 (2)	0.6730 (2)	0.0137 (7)	
H7	0.0396	0.7293	0.6329	0.016*	
C8	0.10854 (11)	0.7181 (2)	0.7431 (2)	0.0099 (6)	
C9	0.14192 (13)	0.6463 (2)	0.8007 (2)	0.0147 (7)	
H9	0.1729	0.6671	0.8491	0.018*	
C10	0.13017 (13)	0.5451 (2)	0.7879 (3)	0.0168 (7)	
H10	0.1528	0.4970	0.8287	0.020*	
C11	0.25859 (12)	0.4103 (2)	0.9057 (2)	0.0109 (6)	
H11	0.2419	0.4740	0.8964	0.013*	
C12	0.31067 (12)	0.4040 (2)	0.9544 (2)	0.0115 (6)	
H12	0.3295	0.4633	0.9803	0.014*	
C13	0.33621 (12)	0.3117 (2)	0.9664 (2)	0.0099 (6)	
C14	0.30682 (11)	0.2278 (2)	0.9260 (2)	0.0111 (6)	
H14	0.3232	0.1639	0.9307	0.013*	
C15	0.25439 (12)	0.2359 (2)	0.8795 (2)	0.0117 (6)	
H15	0.2348	0.1776	0.8536	0.014*	
C16	0.47655 (12)	0.3749 (2)	1.0850 (2)	0.0118 (6)	
H16	0.4989	0.4314	1.0915	0.014*	
C17	0.42538 (12)	0.3837 (2)	1.0310 (2)	0.0115 (6)	
H17	0.4126	0.4459	1.0002	0.014*	
C18	0.39187 (12)	0.3015 (2)	1.0209 (2)	0.0101 (6)	
C19	0.41335 (12)	0.2105 (2)	1.0646 (2)	0.0113 (6)	
H19	0.3920	0.1522	1.0570	0.014*	
C20	0.46497 (12)	0.2047 (2)	1.1181 (2)	0.0109 (6)	
H20	0.4791	0.1426	1.1474	0.013*	

### (III) Poly[[tris(nitrato-κ2O,O')neodymium(III)]-bis(µ-4,4'-bipyridine N,N'-dioxide-κ2N:N'}] Atomic displacement parameters (Å^2^)

**Table:**

	*U*^11^	*U*^22^	*U*^33^	*U*^12^	*U*^13^	*U*^23^
Nd1	0.00565 (7)	0.00592 (7)	0.00960 (7)	−0.00075 (8)	0.00098 (5)	−0.00046 (8)
O1	0.0140 (12)	0.0039 (10)	0.0155 (11)	−0.0026 (9)	0.0018 (9)	0.0000 (9)
O2	0.0142 (12)	0.0030 (10)	0.0215 (12)	−0.0035 (9)	0.0046 (10)	−0.0026 (9)
O3	0.0036 (10)	0.0125 (11)	0.0143 (11)	0.0003 (9)	−0.0023 (9)	−0.0010 (9)
O4	0.0050 (10)	0.0122 (11)	0.0121 (10)	0.0010 (9)	−0.0009 (8)	−0.0004 (9)
O5	0.0326 (16)	0.0244 (14)	0.0197 (13)	−0.0162 (12)	0.0095 (12)	−0.0016 (11)
O6	0.0301 (16)	0.0144 (12)	0.0295 (14)	0.0025 (11)	0.0136 (12)	0.0065 (11)
O7	0.060 (2)	0.0106 (12)	0.0401 (16)	−0.0062 (12)	0.0397 (16)	0.0002 (12)
O8	0.0165 (13)	0.0297 (14)	0.0104 (11)	0.0076 (11)	0.0032 (10)	0.0031 (10)
O9	0.0118 (11)	0.0138 (12)	0.0155 (11)	0.0024 (9)	0.0028 (9)	0.0025 (9)
O10	0.0190 (13)	0.0168 (12)	0.0236 (13)	0.0047 (10)	0.0115 (11)	−0.0020 (10)
O11	0.0140 (11)	0.0103 (12)	0.0153 (11)	0.0011 (8)	0.0021 (9)	0.0004 (8)
O12	0.0122 (12)	0.0097 (11)	0.0117 (11)	0.0000 (9)	−0.0007 (9)	−0.0002 (9)
O13	0.0160 (13)	0.0240 (13)	0.0159 (12)	−0.0041 (10)	−0.0014 (10)	−0.0109 (10)
N1	0.0105 (14)	0.0081 (13)	0.0081 (12)	−0.0017 (10)	0.0013 (11)	−0.0009 (10)
N2	0.0123 (14)	0.0068 (13)	0.0164 (14)	−0.0004 (11)	0.0064 (12)	−0.0015 (11)
N3	0.0085 (13)	0.0107 (13)	0.0076 (12)	0.0021 (11)	−0.0002 (10)	−0.0008 (10)
N4	0.0082 (12)	0.0122 (13)	0.0074 (11)	0.0004 (11)	0.0017 (10)	−0.0018 (10)
N5	0.040 (2)	0.0092 (14)	0.0285 (17)	−0.0060 (14)	0.0242 (16)	−0.0043 (13)
N6	0.0126 (14)	0.0106 (13)	0.0145 (13)	−0.0015 (11)	0.0042 (11)	−0.0008 (11)
N7	0.0104 (14)	0.0132 (14)	0.0144 (13)	−0.0016 (11)	0.0045 (11)	−0.0036 (11)
C1	0.0098 (16)	0.0119 (16)	0.0146 (15)	−0.0011 (13)	0.0021 (13)	−0.0001 (13)
C2	0.0117 (16)	0.0104 (15)	0.0131 (15)	0.0023 (13)	0.0022 (13)	−0.0014 (12)
C3	0.0124 (16)	0.0084 (15)	0.0081 (14)	0.0005 (13)	0.0023 (12)	0.0018 (12)
C4	0.0089 (16)	0.0103 (15)	0.0138 (15)	−0.0004 (12)	−0.0005 (13)	0.0022 (13)
C5	0.0103 (16)	0.0113 (16)	0.0149 (16)	0.0014 (13)	0.0037 (13)	0.0013 (13)
C6	0.0125 (17)	0.0115 (16)	0.0156 (16)	−0.0009 (13)	−0.0021 (13)	0.0002 (13)
C7	0.0120 (16)	0.0099 (15)	0.0156 (16)	0.0010 (13)	−0.0021 (13)	0.0007 (13)
C8	0.0111 (15)	0.0071 (14)	0.0123 (14)	0.0015 (13)	0.0044 (12)	−0.0008 (13)
C9	0.0114 (16)	0.0114 (16)	0.0186 (17)	0.0010 (13)	−0.0004 (14)	−0.0016 (13)
C10	0.0122 (17)	0.0132 (16)	0.0218 (18)	0.0042 (13)	−0.0007 (14)	−0.0001 (14)
C11	0.0112 (16)	0.0071 (15)	0.0138 (15)	−0.0001 (12)	0.0023 (13)	−0.0019 (12)
C12	0.0090 (16)	0.0102 (15)	0.0145 (15)	−0.0021 (12)	0.0019 (13)	−0.0029 (13)
C13	0.0100 (15)	0.0119 (15)	0.0082 (14)	−0.0005 (12)	0.0032 (12)	−0.0004 (12)
C14	0.0103 (14)	0.0093 (14)	0.0133 (14)	0.0008 (14)	0.0024 (12)	0.0004 (14)
C15	0.0136 (15)	0.0074 (14)	0.0129 (14)	−0.0027 (13)	0.0018 (12)	−0.0020 (13)
C16	0.0114 (16)	0.0095 (15)	0.0148 (15)	−0.0003 (12)	0.0039 (13)	0.0032 (13)
C17	0.0100 (16)	0.0099 (15)	0.0141 (15)	0.0021 (12)	0.0026 (13)	0.0012 (12)
C18	0.0096 (15)	0.0126 (16)	0.0094 (14)	0.0022 (12)	0.0049 (12)	0.0001 (12)
C19	0.0113 (15)	0.0098 (16)	0.0135 (14)	−0.0017 (12)	0.0044 (12)	−0.0007 (12)
C20	0.0135 (16)	0.0085 (16)	0.0113 (14)	−0.0001 (12)	0.0041 (13)	0.0002 (11)

### (III) Poly[[tris(nitrato-κ2O,O')neodymium(III)]-bis(µ-4,4'-bipyridine N,N'-dioxide-κ2N:N'}] Geometric parameters (Å, º)

**Table:**

Nd1—O4^i^	2.448 (2)	C1—H1	0.9500
Nd1—O3	2.451 (2)	C2—C3	1.393 (4)
Nd1—O2	2.488 (2)	C2—H2	0.9500
Nd1—O1^ii^	2.526 (2)	C3—C4	1.396 (4)
Nd1—O5	2.555 (2)	C3—C8	1.482 (4)
Nd1—O8	2.573 (2)	C4—C5	1.376 (4)
Nd1—O11	2.585 (2)	C4—H4	0.9500
Nd1—O12	2.597 (2)	C5—H5	0.9500
Nd1—O9	2.615 (2)	C6—C7	1.377 (4)
Nd1—O6	2.640 (2)	C6—H6	0.9500
O1—N1	1.331 (3)	C7—C8	1.399 (4)
O1—Nd1^iii^	2.526 (2)	C7—H7	0.9500
O2—N2	1.335 (3)	C8—C9	1.396 (4)
O3—N3	1.328 (3)	C9—C10	1.383 (4)
O4—N4	1.328 (3)	C9—H9	0.9500
O4—Nd1^iv^	2.448 (2)	C10—H10	0.9500
O5—N5	1.263 (4)	C11—C12	1.371 (4)
O6—N5	1.262 (4)	C11—H11	0.9500
O7—N5	1.238 (3)	C12—C13	1.393 (4)
O8—N6	1.279 (3)	C12—H12	0.9500
O9—N6	1.264 (3)	C13—C14	1.389 (4)
O10—N6	1.227 (3)	C13—C18	1.476 (4)
O11—N7	1.261 (3)	C14—C15	1.375 (4)
O12—N7	1.277 (3)	C14—H14	0.9500
O13—N7	1.237 (3)	C15—H15	0.9500
N1—C5	1.351 (4)	C16—C17	1.370 (4)
N1—C1	1.352 (4)	C16—H16	0.9500
N2—C10	1.347 (4)	C17—C18	1.395 (4)
N2—C6	1.350 (4)	C17—H17	0.9500
N3—C15	1.346 (4)	C18—C19	1.403 (4)
N3—C11	1.358 (4)	C19—C20	1.376 (4)
N4—C16	1.351 (4)	C19—H19	0.9500
N4—C20	1.352 (4)	C20—H20	0.9500
C1—C2	1.375 (4)		
			
O4^i^—Nd1—O3	106.54 (7)	O4—N4—C20	120.2 (2)
O4^i^—Nd1—O2	68.50 (7)	C16—N4—C20	120.6 (3)
O3—Nd1—O2	75.84 (7)	O7—N5—O6	121.4 (3)
O4^i^—Nd1—O1^ii^	73.79 (7)	O7—N5—O5	121.2 (3)
O3—Nd1—O1^ii^	69.19 (7)	O6—N5—O5	117.4 (3)
O2—Nd1—O1^ii^	117.21 (7)	O7—N5—Nd1	168.5 (2)
O4^i^—Nd1—O5	153.95 (8)	O6—N5—Nd1	61.29 (16)
O3—Nd1—O5	66.72 (7)	O5—N5—Nd1	57.42 (15)
O2—Nd1—O5	85.50 (8)	O10—N6—O9	122.5 (3)
O1^ii^—Nd1—O5	122.38 (8)	O10—N6—O8	120.9 (3)
O4^i^—Nd1—O8	133.41 (7)	O9—N6—O8	116.5 (2)
O3—Nd1—O8	82.44 (7)	O13—N7—O11	121.8 (3)
O2—Nd1—O8	153.59 (7)	O13—N7—O12	120.8 (3)
O1^ii^—Nd1—O8	67.03 (7)	O11—N7—O12	117.4 (2)
O5—Nd1—O8	72.07 (8)	N1—C1—C2	120.5 (3)
O4^i^—Nd1—O11	69.68 (7)	N1—C1—H1	119.7
O3—Nd1—O11	166.35 (6)	C2—C1—H1	119.7
O2—Nd1—O11	90.70 (7)	C1—C2—C3	120.8 (3)
O1^ii^—Nd1—O11	120.39 (6)	C1—C2—H2	119.6
O5—Nd1—O11	110.58 (7)	C3—C2—H2	119.6
O8—Nd1—O11	109.88 (7)	C2—C3—C4	117.0 (3)
O4^i^—Nd1—O12	70.06 (7)	C2—C3—C8	122.6 (3)
O3—Nd1—O12	142.80 (7)	C4—C3—C8	120.4 (3)
O2—Nd1—O12	130.40 (7)	C5—C4—C3	120.9 (3)
O1^ii^—Nd1—O12	74.57 (6)	C5—C4—H4	119.6
O5—Nd1—O12	131.16 (7)	C3—C4—H4	119.6
O8—Nd1—O12	75.92 (7)	N1—C5—C4	120.4 (3)
O11—Nd1—O12	49.47 (6)	N1—C5—H5	119.8
O4^i^—Nd1—O9	130.37 (7)	C4—C5—H5	119.8
O3—Nd1—O9	121.01 (7)	N2—C6—C7	120.6 (3)
O2—Nd1—O9	133.70 (7)	N2—C6—H6	119.7
O1^ii^—Nd1—O9	109.03 (7)	C7—C6—H6	119.7
O5—Nd1—O9	67.36 (7)	C6—C7—C8	120.7 (3)
O8—Nd1—O9	49.27 (7)	C6—C7—H7	119.7
O11—Nd1—O9	67.02 (7)	C8—C7—H7	119.7
O12—Nd1—O9	63.80 (7)	C9—C8—C7	117.1 (3)
O4^i^—Nd1—O6	115.92 (7)	C9—C8—C3	121.4 (3)
O3—Nd1—O6	107.00 (7)	C7—C8—C3	121.5 (3)
O2—Nd1—O6	69.17 (7)	C10—C9—C8	120.5 (3)
O1^ii^—Nd1—O6	170.26 (7)	C10—C9—H9	119.8
O5—Nd1—O6	49.03 (8)	C8—C9—H9	119.8
O8—Nd1—O6	103.89 (7)	N2—C10—C9	120.7 (3)
O11—Nd1—O6	65.12 (7)	N2—C10—H10	119.6
O12—Nd1—O6	107.37 (7)	C9—C10—H10	119.6
O9—Nd1—O6	64.68 (7)	N3—C11—C12	120.4 (3)
O4^i^—Nd1—N5	139.01 (8)	N3—C11—H11	119.8
O3—Nd1—N5	88.42 (8)	C12—C11—H11	119.8
O2—Nd1—N5	79.15 (7)	C11—C12—C13	120.7 (3)
O1^ii^—Nd1—N5	146.02 (8)	C11—C12—H12	119.7
O5—Nd1—N5	24.61 (8)	C13—C12—H12	119.7
O8—Nd1—N5	85.48 (8)	C14—C13—C12	117.2 (3)
O11—Nd1—N5	86.77 (8)	C14—C13—C18	120.6 (3)
O12—Nd1—N5	119.07 (7)	C12—C13—C18	122.2 (3)
O9—Nd1—N5	60.38 (7)	C15—C14—C13	121.0 (3)
O6—Nd1—N5	24.79 (8)	C15—C14—H14	119.5
N1—O1—Nd1^iii^	132.26 (17)	C13—C14—H14	119.5
N2—O2—Nd1	129.77 (17)	N3—C15—C14	120.3 (3)
N3—O3—Nd1	129.30 (16)	N3—C15—H15	119.8
N4—O4—Nd1^iv^	134.20 (16)	C14—C15—H15	119.8
N5—O5—Nd1	97.97 (19)	N4—C16—C17	120.9 (3)
N5—O6—Nd1	93.92 (19)	N4—C16—H16	119.5
N6—O8—Nd1	97.58 (17)	C17—C16—H16	119.5
N6—O9—Nd1	95.95 (16)	C16—C17—C18	120.3 (3)
N7—O11—Nd1	97.07 (17)	C16—C17—H17	119.8
N7—O12—Nd1	96.06 (16)	C18—C17—H17	119.8
O1—N1—C5	119.2 (2)	C17—C18—C19	117.3 (3)
O1—N1—C1	120.4 (2)	C17—C18—C13	120.5 (3)
C5—N1—C1	120.4 (3)	C19—C18—C13	122.2 (3)
O2—N2—C10	120.1 (3)	C20—C19—C18	120.6 (3)
O2—N2—C6	119.4 (3)	C20—C19—H19	119.7
C10—N2—C6	120.4 (3)	C18—C19—H19	119.7
O3—N3—C15	119.8 (2)	N4—C20—C19	120.2 (3)
O3—N3—C11	119.8 (2)	N4—C20—H20	119.9
C15—N3—C11	120.4 (3)	C19—C20—H20	119.9
O4—N4—C16	119.2 (2)		
			
Nd1—O3—O4—Nd1^iv^	4.87 (14)	Nd1—O2—O1—Nd1^iii^	91.75 (11)

Symmetry codes: (i) *x*−1/2, −*y*+1/2, *z*−1/2; (ii) *x*, *y*−1, *z*; (iii) *x*, *y*+1, *z*; (iv) *x*+1/2, −*y*+1/2, *z*+1/2.

### (III) Poly[[tris(nitrato-κ2O,O')neodymium(III)]-bis(µ-4,4'-bipyridine N,N'-dioxide-κ2N:N'}] Hydrogen-bond geometry (Å, º)

**Table:**

*D*—H···*A*	*D*—H	H···*A*	*D*···*A*	*D*—H···*A*
C1—H1···O5^v^	0.95	2.61	3.353 (4)	135
C4—H4···O13^vi^	0.95	2.37	3.206 (4)	147
C5—H5···O4^vii^	0.95	2.37	3.163 (4)	141
C9—H9···O9^viii^	0.95	2.63	3.216 (4)	121
C9—H9···O10^v^	0.95	2.58	3.464 (4)	156
C10—H10···O3	0.95	2.30	3.110 (4)	142
C10—H10···O7^viii^	0.95	2.61	3.289 (4)	129
C11—H11···O10^v^	0.95	2.50	3.243 (4)	135
C14—H14···O7^ix^	0.95	2.21	2.998 (4)	139
C15—H15···O1^ii^	0.95	2.31	3.091 (4)	139
C16—H16···O13^v^	0.95	2.56	3.159 (4)	121
C17—H17···O12^v^	0.95	2.37	3.295 (4)	165
C20—H20···O2^iv^	0.95	2.61	3.294 (4)	130

Symmetry codes: (ii) *x*, *y*−1, *z*; (iv) *x*+1/2, −*y*+1/2, *z*+1/2; (v) −*x*+1/2, *y*+1/2, −*z*+3/2; (vi) −*x*, −*y*+1, −*z*+1; (vii) *x*−1/2, −*y*+3/2, *z*−1/2; (viii) *x*, −*y*+1, *z*+1/2; (ix) −*x*+1/2, *y*−1/2, −*z*+3/2.
